# Design and Evaluation of a Chalcone-Based Dimeric Scaffold Targeting Amyloid-Beta Plaques for Early Detection of Alzheimer’s Disease: Synthesis, Radiolabeling, In Vivo Studies, and Integrated Molecular Docking and Dynamics Simulation

**DOI:** 10.34172/apb.46659

**Published:** 2026-05-19

**Authors:** Zahra Ghiamaty, Mehdi Akhlaghi, Safura Jokar, Hooman Hafezi, Omid Bavi, Mohammad Sharifzadeh, Omid Sabzevari, Mehdi Shafiee Ardestani, Tahereh Ghorbandaeipour, Mahboobeh Asadi, Mahshid Kiani, Sara Roustaei, Fatemeh Ebrahimi, Davood Beiki

**Affiliations:** ^1^Department of Nuclear Pharmacy, Faculty of Pharmacy, Tehran University of Medical Sciences, Tehran, Iran; ^2^Research Center for Nuclear Medicine, Tehran University of Medical Sciences, Tehran, Iran; ^3^Department of Mechanical Engineering, Shiraz University of Technology, Shiraz, Iran; ^4^Department of Toxicology and Pharmacology, Faculty of Pharmacy, Tehran University of Medical Sciences, Tehran, Iran; ^5^Toxicology and Poisoning Research Centre, Tehran University of Medical Sciences, Tehran, Iran; ^6^Department of Neurosciences and Addiction Studies, School of Advanced Technologies in Medicine, Tehran University of Medical Sciences, Tehran, Iran

**Keywords:** Alzheimer’s disease, Chalcone, Gallium-68, Molecular imaging, PET/CT imaging, Radiolabeing

## Abstract

**Purpose:**

Amyloid-Beta (Aβ) plaque imaging can serve as a desirable tool for early detection of Alzheimer’s disease (AD). This study presents a new dimer of a chalcone-based scaffold, [68Ga]Ga-DTPA[Ch-DE]2, as a potential radiotracer for Aβ plaques imaging.

**Methods:**

The synthesis of DTPA[Ch-DE]2 and its radiolabeling with gallium-68 were characterized using techniques such as 1H-NMR, mass spectrometry, HPLC, and instant thin-layer chromatography. Neurotoxicity of DTPA[Ch-DE]2 was evaluated using the MTT assay. The stability of radiotracer was tested in various buffer systems and human serum albumin (HSA). The binding affinity of the radiotracer to Aβ aggregations was evaluted on the brain sections from both normal and AD rat models. Positron emission tomography-computed tomography (PET/CT) of brain imaging and Thioflavin S staining characterized the AD model. Biodistribution studies and PET/CT scans were performed on normal and AD rats. Computational studies also were conducted.

**Results:**

[68Ga]Ga-DTPA[Ch-DE]2 was synthesized with over 93% radiochemical purity. It showed high stability in various buffer systems and HSA after 2 hours. The binding assay revealed strong affinity to Aβ42 aggregations. Biodistribution studies and PET/CT imaging indicated favorable brain uptake: 0.54% ID/g and 0.59% ID/g at 2 minutes in normal and AD rats, respectively. Computational studies indicated that DTPA[Ch-DE]2 binds specifically to the Aβ fibrils while maintaining the receptor’s architectural integrity. In vivo studies show that [68Ga] Ga-DTPA[Ch-DE]2 has good brain uptake and retention in AD rats compared with normal.

**Conclusion:**

Thus, results suggest that [68Ga]Ga-DTPA[Ch-DE]2 could be a promising radiotracer for Aβ targeting and early diagnosis of AD.

## Introduction

 Alzheimer’s disease is a progressive and irreversible neurodegenerative disorder with severe loss of memory, intellectual impairment, and behavioral disturbances that interfere with daily life.^1^ It is currently the 6th leading cause of death worldwide among individuals aged over 65 years.^2^ As the most common and deadly form of dementia, in 2022, AD dementia was estimated to affect 32.3 million people globally. This number is expected to increase over time due to the aging population.^3^ With the increasing incidence of AD and its associated mortality, early and definitive diagnosis before the onset of clinical cognitive symptoms is essential to prevent irreversible neurological dysfunction.^4,5^ The two main pathological features of AD are neurofibrillary tangles (NFTs), which consist of hyperphosphorylated tau protein, and extracellular amyloid plaques, primarily composed of Aβ1-40 and Aβ1-42 peptides.^6,7^ According to the reported studies, Aβ plaque deposition begins several years before the clinical symptoms manifest and could be considered one of the key biomarkers for identifying individuals at risk of developing AD.^8,9^ The identification of Aβ plaques using non-invasive nuclear imaging modalities such as PET and single-photon emission computed tomography (SPECT) could provide us with early diagnosis and differentiation of AD from other types of dementia, and the efficacy of anti-amyloid therapeutic interventions.^10–12^

 To date, a various range of radioligands have been developed for targeting and imaging Aβ plaques in AD, including monoclonal antibodies,^13^ peptides,^14,15^ and small molecule derivatives, such as Congo red,^16^ Thioflavin T,^17^ and Chrysamine-G.^18^ Biomolecules typically exhibit limited brain penetration, slow clearance from the bloodstream, and low bioavailability.^19,20^ In contrast, small molecules demonstrate that they could be good candidates for Aβ plaque targeting due to more favorable cerebral pharmacokinetic properties.^21^ A variety of radiolabeled small molecules have been developed for targeting and imaging Aβ plaques in AD individuals. These include [^123^I]IMPY, as a Thioflavin T derivative,^22^ and several PET radiotracers such as ^11^C-Pittsburgh compound B (PIB), which is a 2-phenyl benzothiazole derivative and the first PET radioligand specifically designed for Aβ imaging.^23^ Other PET radiotracers include [^11^C]BF-227,^24^ [^11^C]SB-13,^25^ [^18^F]FDDNP, an amino-naphthyl derivative,^26^ [^18^F]NAV4694 (AZD4694),^27^ [^18^F]GE067 (Flutemetamol/Vizamyl, a PIB analog), [^18^F]AV-45 (Florbetapir/Amyvid), and [^18^F]BAY-94-9172 (Florbetaben/NeuraCeq), which is a stilbene derivative. Among these, Florbetapir, Florbetaben, and Flutemetamol have received FDA approval for clinical use in imaging Aβ plaques in patients with AD.^28–30^ [^125^I]-DMIC was reported as the first chalcone-based Aβ imaging probe by Ono et al in 2007.^31^

 Due to the limited availability of PET cyclotron-based radionuclides such as ^11^C and ^18^F in all nuclear medicine centers, there has been a growing attention toward the application of PET generator-based radionuclides, such as ^68^Ga, for imaging Aβ plaques.^32^ ^68^Ga could be considered as a propitious radionuclide candidate for in vivo β-amyloid plaque imaging due to its advantages such as convenient production and long-term commercial access through a ^68^Ge/^68^Ga generator with a long-lived (270 days), favorable emission properties (β^+^: 89%, E_max_: 1.92 MeV, t_1/2_: 67.7 min), that allow prompt decay and elimination, lowering patient radiation exposure.^33^

 In this study, with the aim to develop a PET Aβ-targeting probe with optimal brain pharmacokinetics, a novel homodimeric chalcone DTPA[Ch-DE]_2_ (6,9-Bis(carboxymethyl)-14-(4-((E)-3-(4-(diethylamino)phenyl)-acryloyl) phenoxy)-3-(2-(2-(4-((E)-3-(4-(diethylamino)phenyl)-acryloyl)phenoxy) ethylamino)-2-oxoethyl)-11-oxo-3,6,9,12- tetraazatetradecanoic acid) with the regarding more affinity to Aβ plaques than the monomeric counterpart, was prepared and radiolabeled with [^68^Ga]GaCl_3 _(Figure 1). We conducted binding affinity studies of [^68^Ga]Ga-DTPA[Ch-DE]_2_ to Aβ aggregations, as well as in vivo biodistribution and imaging studies in both normal and AD rats. Furthermore, molecular docking studies and molecular dynamics simulations were performed to better understand the action mechanism and the interactions involved between DTPA[Ch-DE]_2_ and Aβ1-42 fibril, and monomer structures.

**Figure 1:**
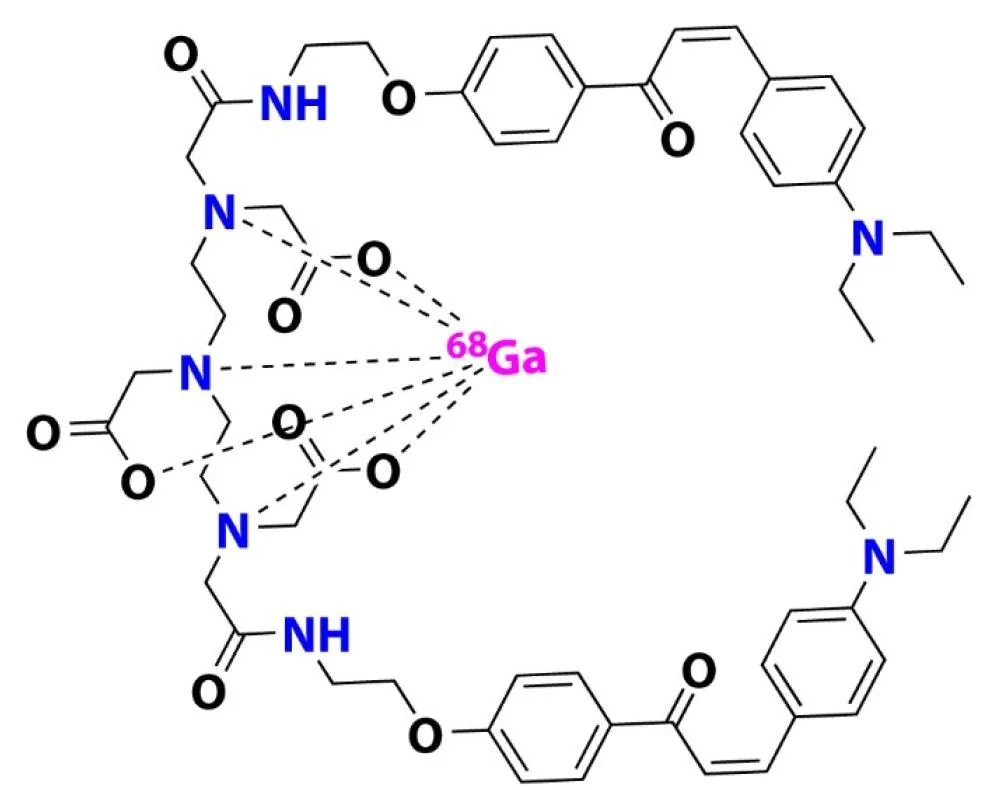
Schematic representation of the suggested structure of [^68^Ga]Ga-DTPA[Ch-DE]_2_

## Materials and Methods

###  Materials 

 All chemicals and reagents were purchased from Sigma-Aldrich (Germany) and Merck and used without any purification. Thin-layer and column chromatography were performed on silica gel (SG) 60 F254 plates (Merck) and silica MN60 (60–200 μm), respectively,. Chromatographic elution solventswere acquired from Amertat Chemical Company. The Aβ1–42 peptide was obtained from Sigma-Aldrich. Ketamine and xylazinewere sourced from Alfasan (Netherlands). A solution of [^68^Ga]GaCl_3_ was eluted from a commercial ^68^Ge/^68^Ga generator (Pars Isotope Co., Karaj, Iran) with an activity of 110 MBq/day using 0.1 M HCl. Male Wistar rats (300-350 g) were supplied by the Royan Research Institute and kept under standard conditions (12-hour light/dark cycle at a room temperature of 20–22 °C) for further operations. The SH-SY5Y cell line was obtained from the Pasteur Institute of Iran.

###  Methods

####  DTPA[Ch-DE]2 Synthesis and Characterization

 DTPA[Ch-DE]_2_ was synthesized Through a six-step reaction (Scheme 1), as previously reported.^34^

**Scheme 1:**
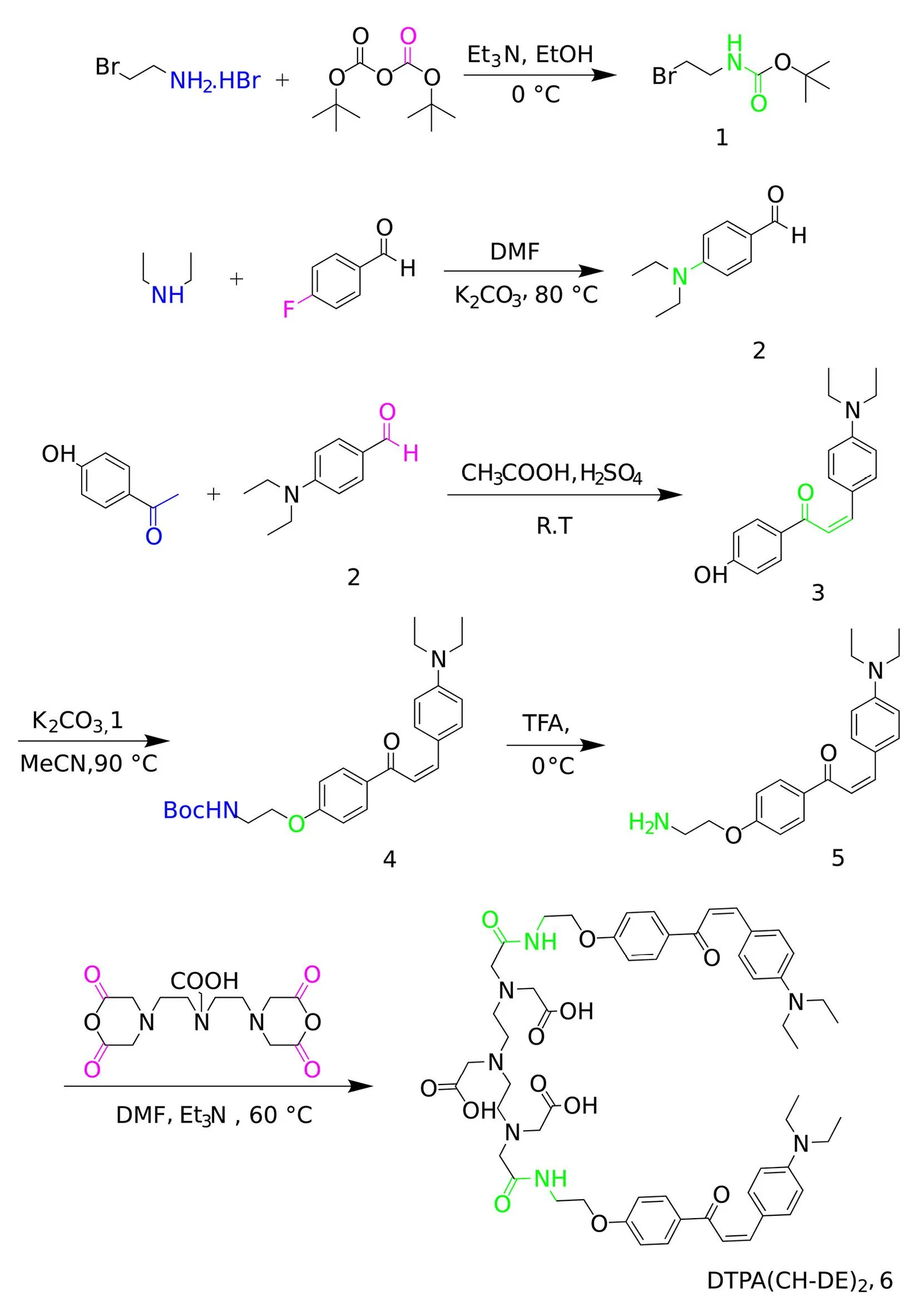
Chemical synthesis of the DTPA[Ch-DE]_2_

####  Synthesis of Tert-Butyl 2-Bromoethylcarbamate (Compound 1).

 A solution of di-tert-butyl dicarbonate (2.6 g, 12 mmol) in 25 mL of ethanol was added dropwise to a stirred mixture of triethylamine (2.76 mL, 20 mmol) and bromoethylamine hydrobromide (2.04 g, 10 mmol) in 5 mL of ethanol while cooling in an ice bath. The reaction mixture was allowed to warm to room temperature and stirred for 15 h after the addition was complete. Then, the solvent was evaporated under reduced pressure to yield a white solid. The solid was subsequently extracted sequentially with water (30 mL) and ethyl acetate (2 × 15 mL). The organic extracts were combined, dried over anhydrous sodium sulfate (Na_2_SO_4_), and the solvent was evaporated to yield compound 1 as a colorless oil (2 g, 90% yield). ^1^H NMR (a Varian INOVA 500 MHz spectrometer, DMSO-*d6*) δ 4. 2 (s, 1H), 3.43 (m, 2H), 3.28 (t, *J* = 6.4 Hz, 2H), 1.37 (s, 9H), (Figure S1).

####  Synthesis of Para-Diethylaminobenzaldehyde (Compound 2).

 Diethylamine (2.9 g, 40 mmol) was first dissolved in 20 mL of Dimethylformamide (DMF) and mixed with potassium carbonate (11.1 g, 80 mmol). Following stirring at 80 °C for 30 min, 4-fluorobenzaldehyde (4.9 g, 40 mmol) was added dropwise, and the reaction was continued at the same temperature for another 12 h. Following completion, the mixture was filtered using a sintered glass funnel, and the resulting solid was washed with ethyl acetate. Extraction was achieved using water and ethyl acetate, then the organic layer was separated, dried over anhydrous Na_2_SO_4_, and concentrated under reduced pressure. The crude product was purified by column chromatography of silica gel using petroleum ether/ethyl acetate as an eluent to afford compound 2 as a yellow solid (4.4 g, 62% yield). ^1^H NMR (a Varian INOVA 500 MHz spectrometer, CDCl_3_) δ 9.70 (S, 1H), 7.70 (d, *J* = 9.1 Hz, 2H), 6.67 (d, *J* = 8.6 Hz, 2H), 3.43 (q, *J* = 7.0 Hz, 4H), 1.21 (t, *J* = 7.1 Hz, 6H), (Figure S2).

####  Synthesis of (Z)-3-(4-(Diethylamino)Phenyl)-1-(4-Hydroxyphenyl) Prop-2-en-1-One (Compound 3).

 A solution of compound 2 (3.54 g, 20 mmol) in acetic acid (60 mL) was dropwise added to a stirring solution of 4-hydroxyacetophenone (2.7 g, 20 mmol) in concentrated sulfuric acid (2 mL). The reaction mixture was stirred at room temperature for 24 hours. It was then transferred into an ice bath, and the pH was adjusted to 7 with 1 M NaOH (Panpeha pH indicator strips, Aldrich Chemical Co., Darmstadt, Germany). The resulting precipitate was filtered and column chromatographed to afford compound 3 as a red powder (4.04 g, 68.5% yield). ^1^H NMR (a Varian INOVA 500 MHz spectrometer, DMSO-*d*_6_) δ 10.28 (s, 1H), 8.01 (d, *J* = 7.7 Hz, 2H), 7.63 (d, *J* = 8.6 Hz, 2H), 7.58 (d, *J* = 8.6 Hz, 2H), 6.87 (d, *J* = 7.7 Hz, 2H), 6.69 (d, *J* = 8.5 Hz, 2H), 3.40 (q,* J* = 7.0 Hz, 4H), 1.11 (t, *J* = 7.0 Hz, 6H), (Figure S3), MS-EI (m/z) (a Agilent G7081B equiped with a quadrupole analyzer): [C_19_H_21_NO_2_] ^+^ [M] ^+^ found: 295.1; [C_18_H_18_NO_2_] ^+^ [M-CH_3_] ^+^ found: 280.1, (Figure S4).

####  Synthesis of (Z)-Tert-Butyl-2-(4-(3-(4-(Diethylamino)Phenyl)Acryloyl)-Phenoxy)Ethylcarbamate (Compound 4).

 To a stirred solution of compound 3 (0.88 g, 3 mmol) in acetonitrile (20 mL), potassium carbonate (2 g, 15 mmol) was added, and stirred at room temperature for 1 h. Afterward, compound 1 (tert-butyl 2-bromoethyl carbamate, 1.55 g, 7.5 mmol) was added dropwise to the reaction mixture and allowed to reflux for 12 hours. The progress of the reaction was checked by TLC. After completion, the solvent was evaporated under a vacuum, and the crude product was purified using silica gel column chromatography, yielding compound 4 as a yellow oily substance (1.03 g, 78.2% yield). ^1^H NMR (a Varian INOVA 500 MHz spectrometer, DMSO-*d6*) δ 8.10 (d, *J* = 8.4 Hz, 2H), 7.65 – 7.57 (m, 4H), 7.05 (d, *J* = 8.5 Hz, 2H), 6.68 (d, *J* = 8.5 Hz, 2H), 4.34 (t, *J* = 8.1 Hz, 1H), 4.07 (t, *J* = 5.8 Hz, 2H), 3.86 (t, *J* = 8.1 Hz, 1H), 3.38 (q, *J* = 7.0 Hz, 4H), 1.39 (s, 9H), 1.10 (t, *J* = 7.0 Hz, 6H), (Figure S5).

####  Synthesis of (Z)-1-(4-(2-Aminoethoxy) Phenyl)-3-(4-(Diethylamino) Phenyl) Prop-2-en-1-One (Compound 5).

 A solution of compound 4 (0.65 g, 1.5 mmol) was prepared in 5 mL of dichloromethane and stirred for 30 min under an inert atmosphere of argon. In the next step, 2 mL of 25% trifluoroacetic acid (TFA) in a dichloromethane solution was added dropwise to the above solution while keeping it in an ice bath at 0 °C. Afterward, the reaction mixture was stirred at this temperature for 1 h, allowed to reach room temperature, and stirred for an additional 12 h. During the work-up, the solvent was evaporated under reduced pressure until dryness. The residue was then purified by silica gel chromatography. Any impure fractions were further purified by recrystallization: the residue was dissolved in 1 mL of methanol and slowly added to 50 mL of cold diethyl ether. The mixture was stirred in an ice bath for 6 h, which resulted in the formation of compound 5 as a yellow pure precipitate (0.31 g, 61.1% yield). ^1^H NMR (Varian INOVA, 500 MHz, DMSO-*d6*) δ 8.12 (d, *J* = 8.5 Hz, 2H), 7.67-7.58 (m, 4H), 7.08 (d, *J* = 8.5 Hz, 2H), 6.69 (d, *J* = 8.5 Hz, 2H), 4.17 (t, *J* = 5.4 Hz, 2H), 3.40 (q,* J* = 7.1 Hz, 4H), 3.10 (t, *J* = 5.5 Hz, 2H), 1.11 (t, *J* = 7.1 Hz, 6H), (Figure S6).

####  Synthesis of 6,9-Bis(Carboxymethyl)-14-(4-((Z)-3-(4-(Diethylamino)Phenyl)-Acryloyl) Phenoxy)-3-(2-(2-(4-((Z)-3-(4-(Diethylamino)Phenyl)-Acryloyl) Phenoxy) Ethylamino)-2-Oxoethyl)-11-Oxo-3,6,9,12-Tetraazatetradecanoic Acid (Compound **6**, DTPA[Ch-DE]_2_).

 Diethylenetriaminepentaacetic acid bisanhydride (64 mg, 0.1 mmol) was dissolved in 8 mL of anhydrous DMF under nitrogen. Subsequently, a solution of compound 5 ((E)-1-(4-(2-aminoethoxy)phenyl)-3-(4-(diethylamino)phenyl)prop-2-en-1-one, 98 mg, 0.3 mmol) in 5 mL of dry DMF was added dropwise to the above solution. The pH of the reaction mixture was adjusted to 8 with triethylamine and stirred gently at 60 °C under nitrogen. The progress of the reaction was monitored using TLC. Upon completion of the reaction, the solvent was removed under reduced pressure, and the crude product was dissolved in a minimum amount of methanol. Precipitation was initiated by adding diethyl ether. The resulting mixture underwent a liquid-liquid extraction using ethyl acetate and water. The aqueous phase was subsequently purified using a C18 reverse-phase column to remove any residual unreacted Diethylenetriaminepentaacetic acid (DTPA) and then eluted with ethanol. After evaporating the solvent, a yellow solid of compound 6 (DTPA[Ch-DE]_2_) was obtained (95 mg, 94.6% yield). The MS-ESI (m/z) (Waters Synapt G1 HDMS equipped with a Q-TOF analyzer) spectrum showed the following peaks: calculated for [C_56_H_71_N_7_Na_3_O_12_]^3+^ [M + 3Na]^3+^ 1102.4837/3 = 367.4945, found: 367.1971 and calculated for [C_56_H_71_N_7_Na_2_O_12_]^2+^ [M + 2Na]^2+^ 1079.4945/2 = 539.7472, found: 540.5312. Fourier-transform infrared (FTIR) spectra were obtained using KBr pellets using a PerkinElmer Spectrum.

###  Radiolabeling of DTPA[Ch-DE]_2_ with ^68^Ga

 To radiolabel DTPA[Ch-DE]_2_ with ^68^Ga, a fresh solution of [^68^Ga]GaCl_3 _was eluted from a sterile^68^Ge/^68^Ga PARS-GalloGEN generator using 0.1 N HCl (3 mL). The [^68^Ga]GaCl_3_ solution was then added to a vial containing 50 μg of DTPA[Ch-DE]_2_ in 420 μL of sodium acetate buffer (0.1 M, pH 7). The reaction mixture was incubated for 10 min at 70-75 °C and pH 4–4.5. The radiolabeled product was purified using a Sep-Pak C18 cartridge (Waters, Milford, MA, USA), which was treated with ethanol and activated with a 30% (v/v) ethanol-water mixture. To remove any free ^68^Ga, the column was rinsed with 5 mL of deionized water. [^68^Ga]Ga-DTPA[Ch-DE]_2_ was eluted using 1 mL of 50% (v/v) ethanol-water mixture. Finally, to ensure sterilization and eliminate any colloidal impurities, the final product was filtered through a 0.22-micron filter (Merck Millipore, Darmstadt, Germany). ITLC (SG, Merck, Darmstadt, Germany) and HPLC (a Agilent 1200 with a C18 column 4.6 × 250 mm, 5 µm coupled with a UV detector λ = 254 nm) were utilized to determine radiolabeling efficiency and radiochemical purity. For ITLC, we employed a 1.5 cm wide silica gel chromatography paper (ITLC-SG), and a 1:1 mixture of methanol and 1 M ammonium acetate as the mobile phase using a TLC scanner (MiniGita, RAYTEST, Germany). Radiochemical purity was assessed by reverse phase HPLC (Agilent 1200 on a C18 column 4.6 × 250 mm, 5 µm) with a gradient elution program, which was equipped with a sodium iodide NaI(Tl) detector (RAYTEST, Straubenhardt, Germany). The mobile phase consisted of water (A) and acetonitrile (B) with 0.1% TFA. The gradient elution was performed in the following sequence: 0-2 min with 100% A, 2-4 min with 25% B, 4-6 min with 50% B, and 6-25 min with 100% acetonitrile, at a flow rate of 1 mL/min.

###  Determination of Partition Coefficient (Log p)

 To determine the n-octanol/PBS partition coefficient (Log P), the radiotracer (100 μL, 3.7 MBq) was added to a vial containing 1.5 mL of n-octanol and 1.5 mL of PBS (0.1 M, pH 7.4). The mixture was vigorously vortexed at room temperature for 10 min and then centrifuged at 3500 rpm for 5 min to achieve complete phase separation. Equal volumes of each phase (500 µL) were then measured for radioactivity using a gamma counter. Log P was determined as the logarithmic ratio of n-octanol phase counts to PBS phase counts.

###  In-Vitro Stability of Radiotracer 

 The stability of [^68^Ga]Ga-DTPA[Ch-DE]_2_ was evaluated by incubating the radiotracer (100 μL, 3.7 MBq) at 37 °C in separate vials containing 1 mL of different solutions: 0.1 M PBS (pH 7.4), normal saline (pH 5.5), and HSA. To test serum stability, blood was drawn from a healthy volunteer and allowed to clot at 37 °C for 45 min. The radiochemical purity was evaluated at different times (15 to 120 min) using TLC.

###  Cytotoxicity Assay

 The neurotoxicity of the synthesized chalcone was evaluated on the SH-SY5Y human neuroblastoma cell line, as previously reported.^35^ The SH-SY5Y cells were seeded in 96-well plates at a density of 3 × 10^4^ cells/well and incubated for 24 h. Following 24 h, the culture medium was refreshed with a new medium consisting of various concentrations of the chalcone (a range from 5 to 250 μg/mL), and incubated for 24 h and 48 h at 37 °C. After incubation, the culture medium was removed, and 50 μL of MTT solution (0.5 mg/mL in PBS) was added to each well and allowed to incubate for 4 h at 37 °C in the dark. The resulting crystals were dissolved in 100 μL DMSO and gently shaken for 15 min. The absorbance was recorded at 570 nm using a microplate reader (INFINITE M1000).

###  In-Vitro Amyloid Binding Assay 

 For Aβ1-42 aggregation preparation, 0.1 mg lyophilized peptide was dissolved in 1 mL of phosphate buffer solution (10 mM sodium phosphate, 1 mM EDTA, pH 7.4) and incubated at 37 °C for 42 h with shaking. For binding assays, PBS (900 μL), Aβ1-42 aggregates (30 nM, 100 μL), and different radiotracer concentrations (0.5–100 nM, 100 μL) were incubated for 2 h at room temperature in borosilicate glass vials. Nonspecific binding was defined by co-incubation using a 100-fold molar excess of nonradioactive chalcone as a blocking agent. Samples were filtered through filters 0.22 μm, washed with 5 mL of 10% ethanol to remove the free radiotracer, and the remaining activity on the filters was counted with a gamma counter. Specific binding was determined by the subtraction of total from nonspecific binding. The maximum binding capacity (B_max_) and dissociation constant (K_d_) were determined by nonlinear regression using GraphPad Prism 9.5.1.

###  AD Model Development and Characterization

 The animal studies were performed under national guidelines for animal research and recived approval Code: IR.TUMS.TIPS.REC.1398.172 from the ethical committee at Tehran University of Medical Sciences Research.

 The AD model was induced in Male Wistar rats (300–350 g) through stereotaxic surgery with a stereotaxic apparatus (Stoelting, USA).^36^ The rats were anesthetized by intraperitoneal injection of a ketamine/xylazine mixture (70 mg/kg and 10 mg/kg, respectively) and positioned in a stereotaxic frame. The dorsal scalp was shaved and incised to expose the skull. After cleaning, the Bregma and Lambda landmarks were identified, and the stereotaxic coordinates of the CA1 region of the hippocampus were determined according to the Paxinos and Watson rat brain atlas: anteroposterior (AP) = -3.2 mm, mediolateral (ML) = + 1.5 mm, and dorsoventral (DV) = -2.7 mm.^37^ 2 μL of aggregated Aβ (1 μg/side) or PBS (sham group) was injected intracerebrally into all rats using a Hamilton microsyringe. The incision was then sutured, and animals were kept under standard laboratory conditions (25 °C, 12 h light/dark cycle) in the Comprehensive Research Laboratory of Tehran University of Medical Sciences for further evaluation. To confirm AD induction, the cerebral glucose metabolism of the animals was evaluated using FDG-PET imaging. Briefly, the animals were fasted for 24 h before imaging and then injected with 26 MBq of FDG in 250 μL of normal saline via the tail vein. After a 45-minute uptake period, the animals were anesthetized, and PET scans were obtained over a 10-minute interval. Moreover, the presence of Aβ aggregations in the brain tissues of the animals was assessed using Thioflavin S Staining. The brains were excised and fixed with 10% formaldehyde following cardiac perfusion to complete depletion of blood. The fixed tissues were sectioned into 5 μm slices by a microtome. The slides were incubated in a 0.5% Thioflavin S solution prepared in 0.1 N hydrochloric acid (Sigma, England) for 10 minutes in a humid, dark chamber. Afterward, they were stained and then rinsed with 50% ethanol, followed by distilled water. The Aβ plaques were then viewed under an Olympus IX71 fluorescence microscope (Japan).

###  MicroPET Imaging of Brain Tissue 

 To assess the presence of Aβ aggregations and binding affinity of the [^68^Ga]Ga-DTPA[Ch-DE]_2_ to them, the brain sections from both normal and AD rat models with a thickness of 5 µm were prepared using a microtome and fixed on glass slides. The brain sections were incubated with the radiotracer (100 μL, 3.7 MBq) for 30 minutes at room temperature. Following incubation, any free and nonspecifically bound radiotracers were removed by washing the sections with 40% ethanol and then deionized water. The brain sections were subsequently dried and imaged using microPET (Xtrim PET, Parto Nefar Persia Co., Iran) for 15 min.

###  Biodistribution Studies and PET/CT Imaging

 The biodistribution studies of the [^68^Ga]Ga-DTPA[Ch-DE]_2_ were performed in AD and normal rats to evaluate the brain uptake, excretory pathways, and accumulation in non-target organs. The radiotracer (9.25 MBq, 250 µL) was injected into all of the animals via the tail vein. The animals were euthanized 2, 30, and 60 min post-injection. The target organs were then removed, rinsed with cold saline, weighed, and counted with a γ-counter. The results were expressed as the percentage of injected dose/gram (% ID/g). Additionally, the uptake in the removed organs was assessed using PET imaging with a 10-minute acquisition and subsequent drawing of regions of interest (ROI).

 A whole-body PET/CT scan of AD and normal rats was performed using a PET/CT scanner (Biograph 6, Siemens Medical Solutions, Erlangen, Germany). The animals were intravenously injected with [^68^Ga]Ga-DTPA[Ch-DE]_2_ and florbetapir (9.25 MBq, 250 μL) as a reference radiotracer, and anesthetized using a ketamine/xylazine mix. Dynamic PET imaging was then acquired for about 20 minutes on the prone-positioned animals. CT scanning was also performed to localize anatomy and attenuation correction (20 seconds, 80 kV, 150 mAs, 1.25 mm spatial resolution). PET images were reconstructed using a filtered back-projection algorithm and co-merged with match-associated CT images.

###  Statistical Analysis

 All values were reported as mean ± standard deviation (SD) and statistical analysis was performed by student t-test or one-way ANOVA using GraphPad Prism 9.5.1 software. Values ​​of *P* < 0.01, *P* < 0.001, and *P* < 0.0001 were considered statistically significant in all experiments.

###  Molecular Docking 

 To perform molecular docking studies, the 3D structure of the Aβ1-42 fibril (code: 2BEG) and Aβ1-42 monomer (code: 1IYT) was taken from the website RSCB (http://www.rcsb.org/). Briefly, some processes were performed on the 3D structures, including the deletion of water molecules and other isoforms using UCSF Chimera software, adding hydrogen atoms, determination of bond orders, optimization, and energy minimization with the GROMOS96 implementation by Swiss-PDB Viewer version 19 3.7 software. The 2D structures of DTPA[Ch-DE]_2_ and florbetapir, PIB compound as the reference ligands, were prepared by ChemSketch software and minimized using the HyperChem 8.0 program with molecular mechanics (RMSD 0.01 Å and AMBER force field) and semi-empirical (PM3) techniques. Subsequently,they were saved in pdb format. Finally, polar hydrogen atoms and charge were added to their structures in order to optimize the energy of the compounds and were saved in pdbqt format using the Autodock tool software.

 Molecular docking was performed using Autodock Vina software to obtain the optimized conformations.^38^ Briefly, DTPA[Ch-DE]_2_ and florbetapir, PIB compound were docked on the 3D structure of the Aβ1-42 fibril and Aβ1-42 monomer with gid boxes dimensions of 126 Å, 80 Å and 88 Å, grid spacing 0.375 Å, and coordinate X = -1.306, Y = 0.028, and Z = 1.083 for the Aβ1-42 fibril and monomers. AutoDock Tools and LigPlot ^+^ were used to visualize the ligand-target interactions.

###  Molecular Dynamics Simulation

 The molecular dynamics (MD) simulations were performed using NAMD 3.0, leveraging GPU acceleration and the CHARMM36 force field to model atomic interactions. Trajectory visualization and analysis were performed with Visual Molecular Dynamics (VMD), following established methodologies.^39^ The 3D structure of DTPA[Ch-DE]₂ was built using ChemSketch and optimized at the PM3 semi-empirical level in HyperChem. CHARMM36-compatible parameters were generated using the CHARMM General Force Field (CGenFF) program (version 4.6) via the CGenFF web interface. Missing bond, angle, dihedral, and improper parameters were assigned by analogy with chemically similar fragments within CGenFF. Penalty scores were all below 20, indicating good parameter quality. Each receptor was embedded in a TIP3P water box (dimensions: 81 × 61 × 67 Å) and subjected to energy minimization via the steepest descent method.^40,41^ System equilibration was confirmed by monitoring the RMSD and total energy, which reached stable values after 100 ns. For robust statistical analysis, three independent 100-ns simulations were run for each receptor-ligand complex, and data were averaged over the equilibrated trajectory segments. Ligands were incorporated into pre-equilibrated receptor structures, after which the systems underwent further energy minimization and equilibration.

 Temperature control (310 K) was achieved with a Langevin thermostat, while pressure was stabilized using a Nosé-Hoover barostat. Electrostatic interactions at long range were evaluated via the PME method, applying a 12 Å cutoff distance and 1 Å grid spacing.^42^ Analytical assessments utilized trajectory data from the final 100 ns. Binding free energies and interaction forces were derived using a custom Tcl script, while structural stability was evaluated through the Radius of Gyration (RoG) and Root Mean Square Fluctuation (RMSF) measurements. These metrics offered insights into receptor compactness, flexibility, and conformational dynamics during ligand interactions.^5,43^

## Results and Discussion

 Alzheimer’s disease is considered the most prevalent form of dementia, with approximately 55–60% of all dementia cases in the elderly. Regarding the increasing 48.2% risk of AD in individuals over 95 years old, without any potential cure, there will be socio-economic concerns for the healthcare system in the future.^44^ Early diagnosis of AD could result in potentially therapeutic interventions and slowing down the disease process, and reducing the social and economic burden on healthcare systems.^4,5^ With this aim, numerous radiotracers have been evaluated for targeting and imaging of Aβ plaques. Small organic molecules, among them, are promising tracers for imaging Aβ owing to their preferred properties.^21,28–30^ Chalcones have several mechanisms against AD, including targeting and binding tightly to Aβ plaques, and inhibition of Aβ fibril deposition and the action of enzymes such as acetylcholinesterase (AChE), butyrylcholinesterase (BuChE), and pseudocholinesterase.^45-48^ These multifaceted interactions contribute to the development of the chalcone-based scaffolds as a promising strategy for the diagnosis and therapy of Alzheimer’s disease.

###  Synthesis of DTPA[Ch-DE]2 

 It has been illustrated in previous studies that the introduction of an electron-donating group at the R_2_ position of chalcone derivatives was observed to increase their binding affinity against Aβ plaques (Scheme 2).^31,49^ Additionally, the introduction of various aromatic substituents at the R_2_ position has been observed to enhance lipophilicity, thereby providing improved permeability across the BBB. Since planarity of the ligand is a critical requirement for fitting within the hydrophobic pockets of the β-sheet structures of Aβ plaques, the incorporation of aromatic groups in the chalcone backbone ensures the retention of this planar conformation for optimal interaction.^50^,^51^

**Scheme 2:**
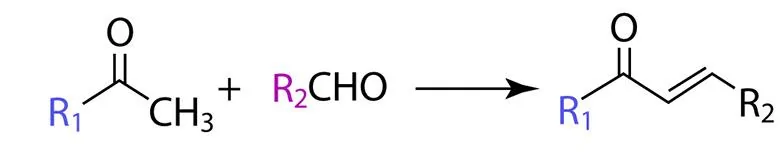
Chalcone synthesis via aldol condensation reaction

 Based on this concept, we designed DTPA[Ch-DE]_2_, a scaffold consisting of a dimer of chalcone with a core DTPA, which acts as a chelator for radiolabeling with [^68^Ga]GaCl_3_. This design aims to enhance the binding affinity to Aβ plaques. Molecular docking and MD simulation studies demostrated that the presence of two chalcone units symmetrically positioned on either side of the DTPA molecule could improve binding of the compound to several sites along the β-sheet regions of Aβ aggregates. Besides, The chalcone derivative synthesized in our study (DTPA[Ch-DE]_2_) has a higher binding affinity to amyloid beta plaques than previous similar works (DTPA[Ch-DM]_2_),^34^ as evident from docking studies in vitro test results (binding affinity energy (kcal/mol): -5.7 and -5.2 for DTPA[Ch-DE]_2 _and DTPA[Ch-DM]_2_, respectively. In this study, we strategically introduced an aromatic group with an electron-donating substituent at position R_2_ and a phenolic group at position R_1_ to facilitate subsequent amination and conjugation to DTPA.

 The DTPA[Ch-DE]_2_ compound was successfully synthesized and purified by RP HPLC to yield a yellow solid with a purity of greater than 95%, as presented in Figure S7. The molecular identity of the DTPA[Ch-DE]_2_ was determined by mass spectrometry. The peaks found at m/z: 540.53 ([M + 2Na]^2+)^ and m/z: 367.19 ([M + 3Na]^3+)^ in the mass spectrum confirm the expected molecular structure (Figure S8). Moreover, comparative IR spectroscopy conducted before and after DTPA conjugation confirmed successful chelation, as evidenced by spectral shifts (Figure S9). In the IR spectrum of compound 6, the broad absorption band observed at 3427.6 cm^-1^ corresponds to the O–H stretching vibrations of the carboxylic acid functional group. Additionally, the broadband at 1739.6 cm^-1^ is attributed to the stretching vibrations of the carbonyl group of the carboxylic acid moiety within the compound’s structure. Furthermore, a band at 1623.5 cm^-1^ can be assigned to the amide carbonyl (C = O) stretching vibrations. These spectral features confirm the successful conjugation of compound 5 to the DTPA chelator.

###  Radiolabelling of DTPA[Ch-DE]2 with [68Ga]GaCl3

^68^Ga was selected as an appropriate PET radioisotope for radiolabeling DTPA[Ch-DE]_2 _due to its desirable physicochemical characteristics and convenient availability through the ^68^Ge/^68^Ga generator, in contrast to ^18^F, which is produced using a cyclotron. In vivo stability of metal complexes and the lack of metal ion release has a vital role in the selection of a chelator. According to the Hard and Soft Acids and Bases (HSAB) principle, Ga^3+^ is considered as a hard Lewis acid and tends to form more stable complexes with hard Lewis bases, such as nitrogen atoms in amines and hydroxyl groups in carboxylic acids.^52^ Additionally, the timing of the radiolabeling reaction is crucial because of the short half-life of ^68^Ga. Studies have shown that linear chelators have a faster complexation rate compared to cyclic analogs due to their structural flexibility and improved reaction kinetics, which allows for the formation of neutral complexes that can permeate the BBB.^53,54^ DTPA is an effective octadentate acyclic chelator most commonly used in chelation chemistry. It is efficiently capable of coordinating Ga^3+^ under mild conditions to form thermodynamically stable complexes.^53^

 The optimal condition for radiolabeling was evaluated under various temperatures (R.T. to 90 °C) and pH conditions (1 to 7). The best efficiency of radiolabeling was achieved at a temperature of 70–75 °C and a pH range of 4.0–4.5. Under these optimum conditions, [^68^Ga]Ga-DTPA[Ch-DE]_2_ was obtained with a radiochemical yield of 87%, a molar activity (Am) of 13.07 MBq/nmol, and radiochemical purity of more than 93%, which was achieved by purification on a Sep-Pak C18 column. As shown in the HPLC chromatogram, the retention times are 0.87 and 20 min for free ^68^Ga and [^68^Ga]Ga-DTPA[Ch-DE]_2_, respectively (Figure 2A). Moreover, ITLC data shows retention factors (R_f_) at 0.1 and 0.7, which are related to colloid forms of ^68^Ga and [^68^Ga]Ga-DTPA[Ch-DE]_2_, respectively (Figure 2B).

**Figure 2:**
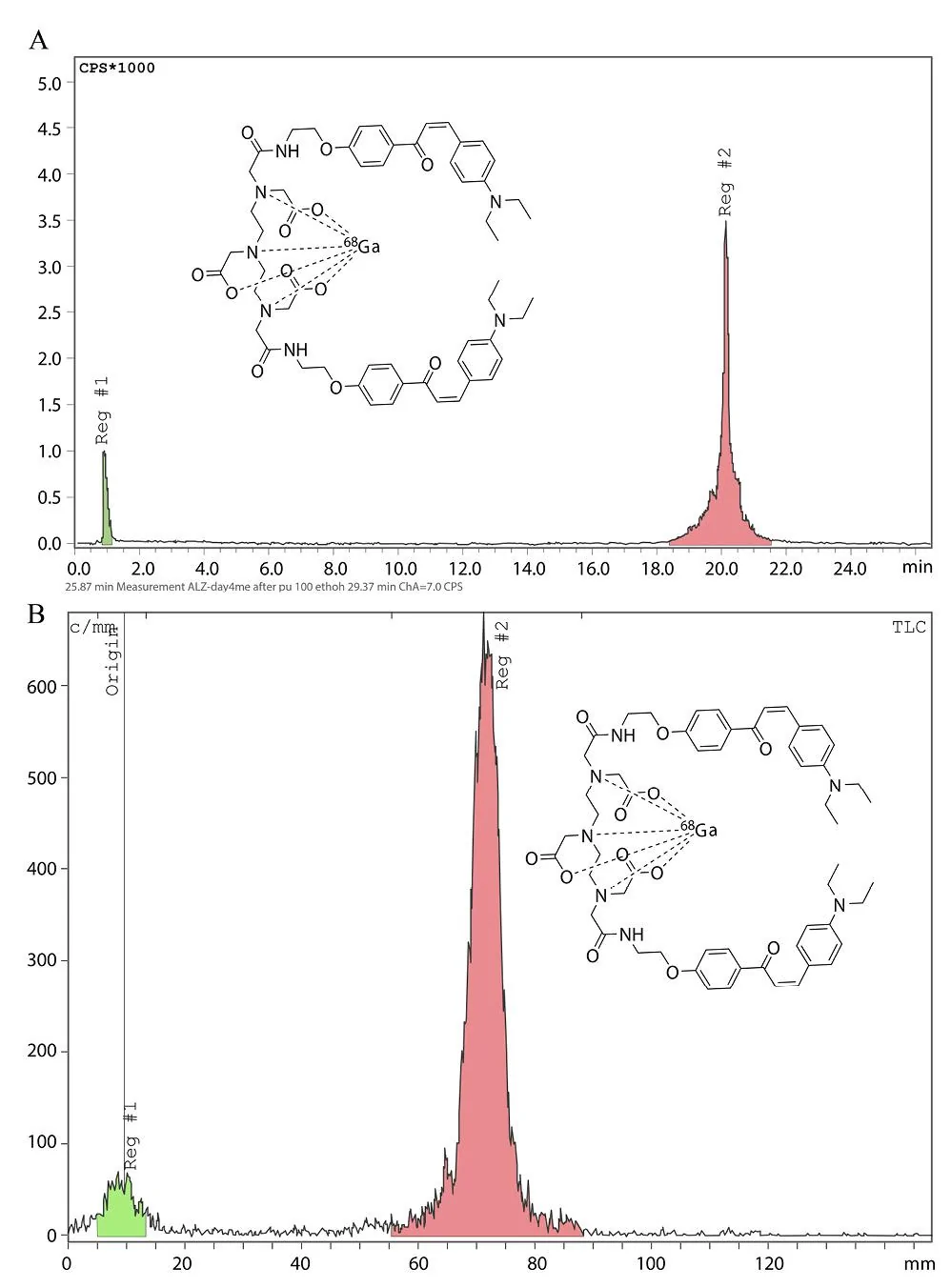
Radiochemical purity of [^68^Ga]Ga-DTPA[Ch-DE]_2_; **A**) HPLC profile of [^68^Ga]Ga-DTPA[Ch-DE]_2_; the peaks at 0.87 and 20 min are related to free ^68^Ga and [^68^Ga]Ga-DTPA[Ch-DE]_2_, respectively.** B**) ITLC analysis, retention factors at 0.1 and 0.7 are for colloid forms of ^68^Ga and [^68^Ga]Ga-DTPA[Ch-DE]_2_, respectively

###  n-Octanol/PBS Partition Coefficient (Log P)

 The lipophilicity of [^68^Ga]Ga-DTPA[Ch-DE]_2_ was obtained using measurement of its n-octanol/PBS (pH 7.4) partition coefficient at room temperature. The resultant log P value was calculated as 2.05 ± 0.14 (mean ± SD, n = 3), which demonstrates a lipophilic nature of ^68^Ga-DTPA[Ch-DE]_2. _Since the optimal window of lipophilicity (log P) of the brain radiotracers is typically 1.0 to 3.0, which is a criterion to effectively penetrate the BBB,^15^ The [^68^Ga]Ga-DTPA[Ch-DE]_2 _could be a good radiotracer for brain imaging.

###  Stability Studies 

 The stability of [^68^Ga]Ga-DTPA[Ch-DE]_2_ was evaluated in normal saline (pH 5.5), PBS (0.1 M, pH 7.4), and human serum at 37 °C for 120 min. The radiochemical purity was monitored at various time intervals using ITLC-SG with a 1:1 mobile phase consisting of 1 M ammonium acetate and methanol. The radiotracer demonstrated high stability (> 90%) in saline, PBS, and human serum within the 120-min period (Figure 3A and 3B).

**Figure 3:**
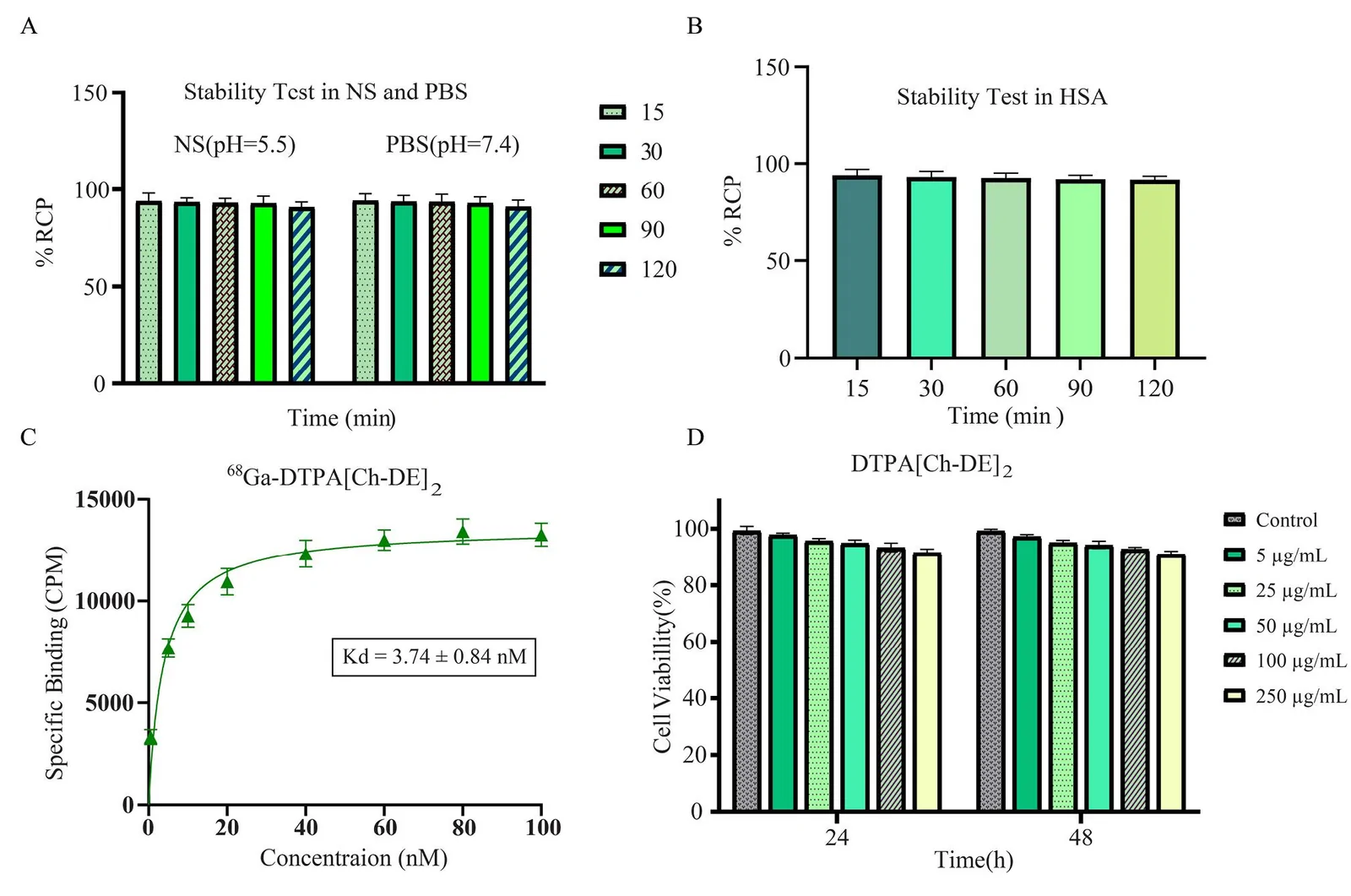
Stability studies of [^68^Ga]Ga-DTPA[Ch-DE]_2_ in **A**) PBS (pH = 7.4), NS (pH = 5.5), and **B**) HSA at 37°C up to 120 min; **C**) Saturation curve for binding of [^68^Ga]Ga-DTPA[Ch-DE]_2_ to Aβ aggregation; **D**) Cytotoxicity assay of DTPA[Ch-DE]_2_ on the SH-SY5Y neuroblastoma cell line after 24 hours and 48 hours of incubation. Cells were treated with 5, 25, 50, 100, and 250 µg/ml of DTPA[Ch-DE]_2_, and cell viability was evaluated by using the MTT test. (mean ± SD, n = 3)

###  In-Vitro Binding Assay

 The binding affinity of [^68^Ga]Ga-DTPA[Ch-DE]_2_ to Aβ aggregation was evaluated through performing total and nonspecific binding studies of [^68^Ga]Ga-DTPA[Ch-DE]_2_ on Aβ peptide aggregation. The values of K_d_ and B_max_ were obtained as 3.74 ± 0.84 nM and 13649 ± 460.49 bound molecules/plaque, respectively (Figure 3C). The binding affinity of [^68^Ga]Ga-DTPA[Ch-DE]_2_ falls within the previously reported Kd range of 3.4–6.5 nM for the chalcone-derived Aβ-targeting agents,^45^ which confirms the strong binding affinity of [^68^Ga]Ga-DTPA[Ch-DE]_2_ to Aβ aggregation.

###  Cytotoxicity Assay

 The cytotoxicity of DTPA[Ch-DE]_2_ was investigated against the SH-SY5Y neuroblastoma cell line at concentrations ranging from 5 to 250 µg/mL for 24 and 48 h after incubation using the MTT assay. As displayed in Figure 3D, no statistically significant cytotoxic effects were observed in the SH-SY5Y cell line at concentrations lower than 250 µg/ml. Meanwhile, the concentration of DTPA[Ch-DE]_2 _used in each injection dose of animal studies is much lower than these values.

###  Animal Models Development

 After injection of Aβ1-42 aggregation into the hippocampus region of animals, the Alzheimer’s model was confirmed using in vivo brain imaging with [^18^F]-FDG-PET to assess changes in glucose metabolism. Additionally, in vitro Thioflavin S staining on brain tissue sections from both AD and normal rats was performed to visualize Aβ aggregation. The Thioflavin S staining confirmed the presence of Aβ fibril aggregation within the AD model compared to normal animals, the Alzheimer’s group revealed 86.3% plaque staining, whereas the normal revealed merely 19.4% (*P* < 0.0001, Figure 4A and 4B). Furthermore, the results of [^18^F]-FDG-PET imaging demonstrate that there is reduced brain uptake of FDG in the Alzheimer’s model compared to the normal animals (Figure 4C and 4D), which confirmed AD induction.

**Figure 4:**
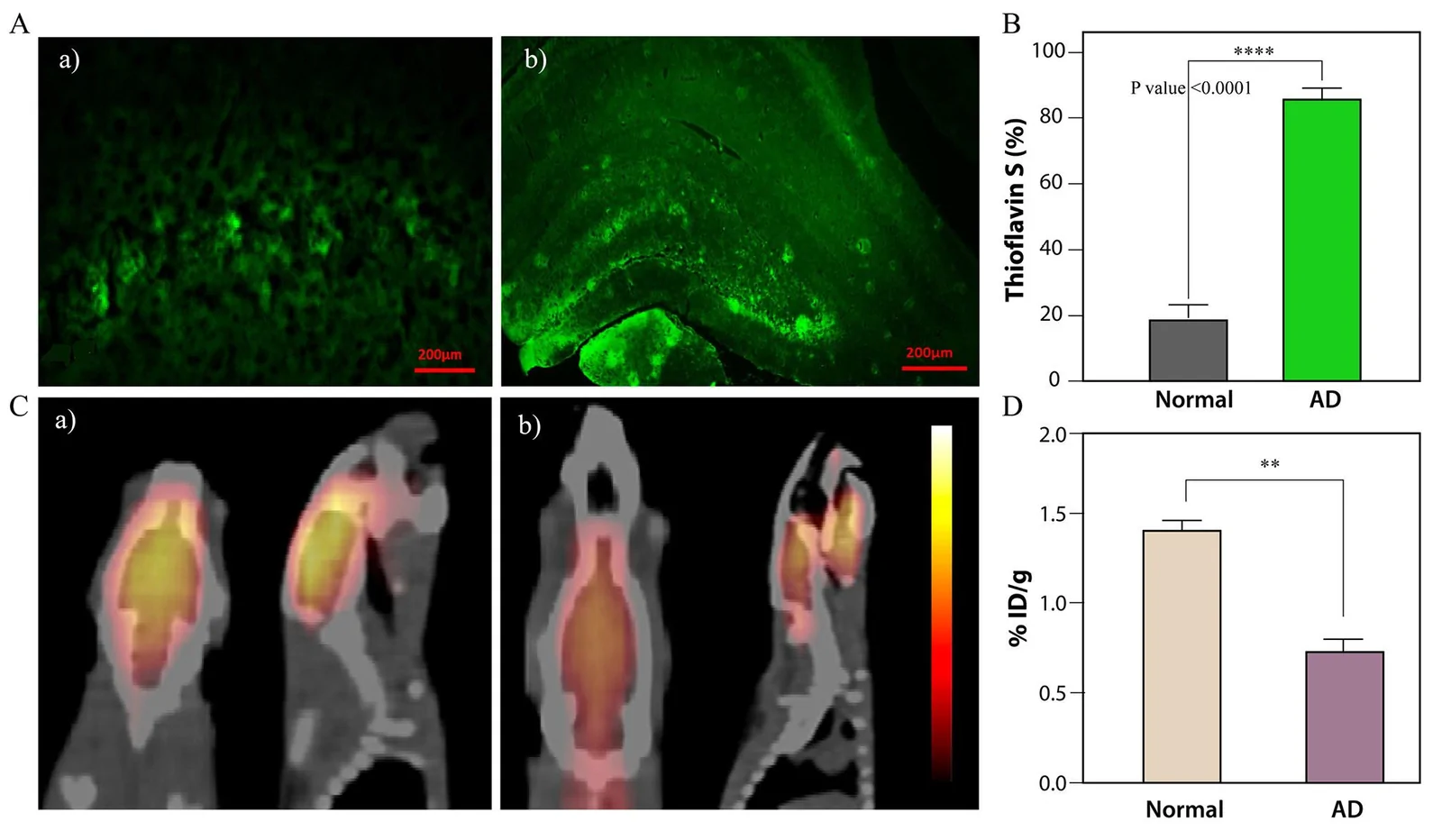
**A**) Thioflavin S staining of Aβ plaques in rat brain tissue sections from Hippocampus **a**) normal **b)** AD; **B**) Statistical analysis of thioflavin S% bound to Aβ plaques between normal and AD groups (n = 3); While *P* < 0.0001 indicates a significant difference in the intensity of thioflavin staining between the Alzheimer’s and normal groups. **C**) [^18^F]FDG-PET/CT images taken in sagittal and coronal views in **a**) normal, **b**) AD rat; **D**) Brain uptake of radioactivity (%ID/g) after injection of [^18^F]FDG in normal and AD rats

###  MicroPET imaging of Brain Tissue Sections

 To further validate the presence of Aβ aggregations and binding affinity of [^68^Ga]Ga-DTPA[Ch-DE]_2_ to Aβ plaques, ex vivo microPET imaging was performed on brain sections from AD and normal rats using microPET after 30-min incubation with radiotracer. The findings showed a significant accumulation of [^68^Ga]Ga-DTPA[Ch-DE]_2 _in brain tissues of Alzheimer’s rats compared to normal rats (Figure 5).

**Figure 5:**
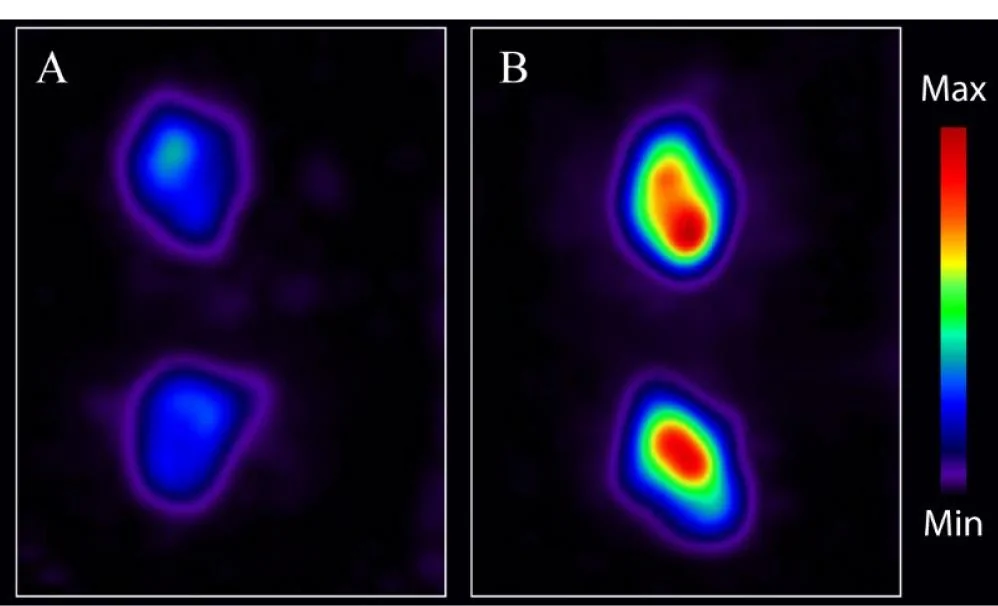
MicroPET images of [^68^Ga]Ga-DTPA[Ch-DE]_2_ in **A**) normal, and **B**) AD rat brain sections

###  Biodistribution and Imaging Studies

 According to the obtained in vitro findings, we are encouraged to perform biodistribution studies of [^68^Ga]Ga-DTPA[Ch-DE]_2_ in AD and normal rats. The biodistribution studies of [^68^Ga]Ga-DTPA[Ch-DE]_2 _in normal and AD rats were performed at 2, 30, and 60 min post-injection. As shown in Figure 6, there is a higher brain uptake and slower washout rate in AD rats than in normal rats. The brain uptake of ^68^Ga-DTPA[Ch-DE]_2_ was similar in both groups, with values of 0.54 ± 0.34% and 0.59 ± 0.55% ID/g at 2 min post-injection for normal and AD rats, respectively. However, over time, the [^68^Ga]Ga-DTPA[Ch-DE]_2_ was washed out rapidly from the brain of normal rats than from ADs due to the presence of Aβ plaques. There is a significant difference (*P* < 0.0001) in the amount of brain uptake of ^68^Ga-DTPA[Ch-DE]_2_ at 30 and 60 min post-injection between two groups. The brain uptake of [^68^Ga]Ga-DTPA[Ch-DE]_2 _after 30 min (0.26 %ID/g) is comparable with other chalcone-derived radiotracers previously reported, such as [^68^Ga]-DT(Ch)_2_ (1.24 %ID/g)_,_^34^ [^99m^Tc]-DT(Ch)_2 _(1.16 %ID/g)_,_^55^ [^18^F]-DMFC (4.43 %ID/g), [^18^F]-MC (5.42 %ID/g), [^125^I]-DMIC (2.43 %ID/g), [^125^I]-IMC (3.52 %ID/g).^56^ Moreover, an initial high uptake was observed in several organs, including the liver, kidneys, lungs, and intestines. Furthermore, the brain uptake of [^68^Ga]Ga-DTPA[Ch-DE]_2_ in normal rats was obtained at 2 and 30 minutes post-injection and was compared with the brain uptake of [^18^F]-Florbetapir as reported in previous studies (Table 1).

**Figure 6:**
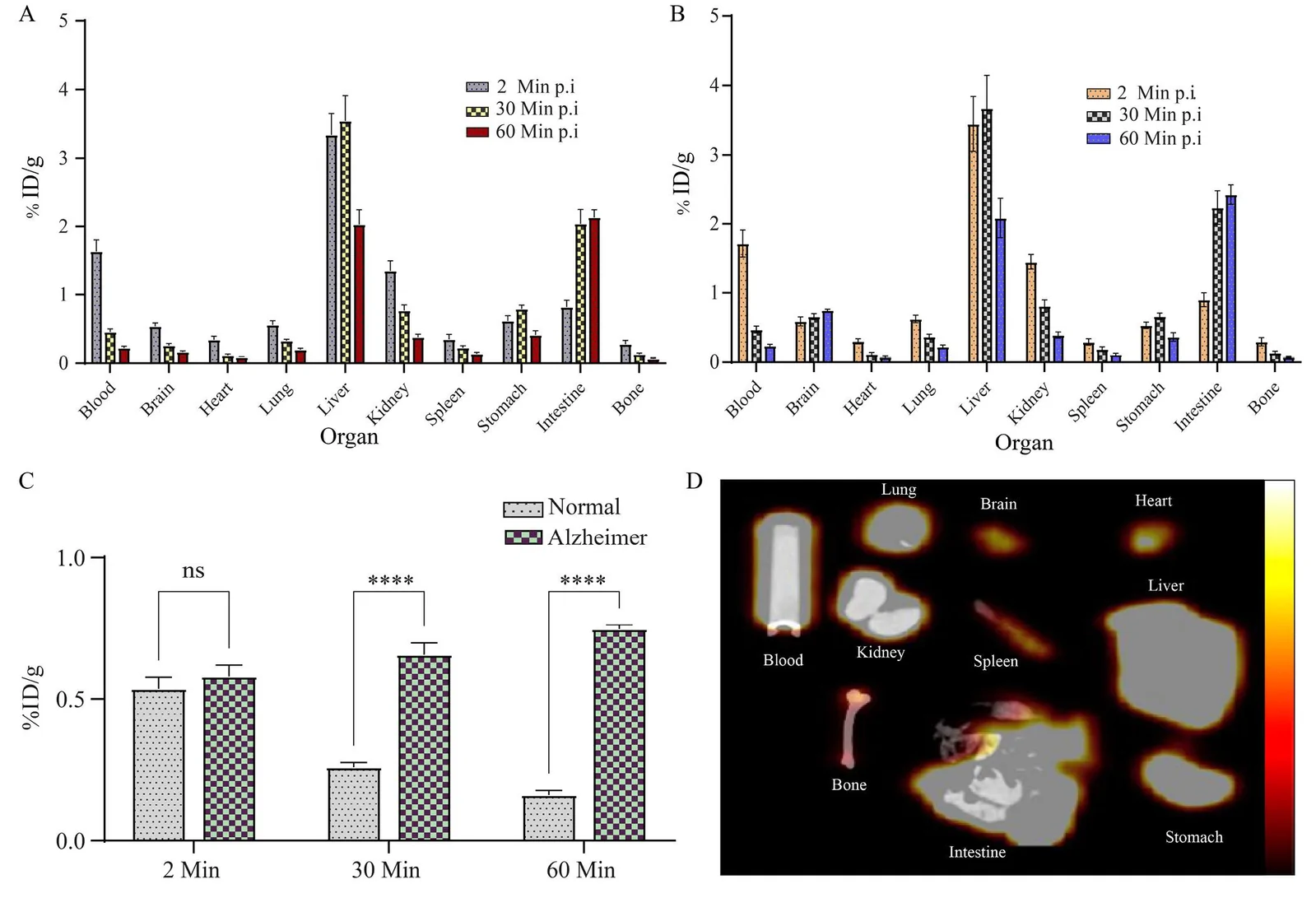
In-vivo biodistribution studies of [^68^Ga]Ga-DTPA[Ch-DE]_2 _(as %ID/g, Mean ± SD, n = 3) in **A**) normal rat, **B**) AD rat, **C**) Statistical analysis of the brain uptake of [^68^Ga]Ga-DTPA[Ch-DE]_2 _between normal and AD rats at 2, 30, and 60 min post-injection, and **D**) PET/CT images of the removed organs from the AD rat after 30 min post-injection

**Table 1:** Brain uptake of [^68^Ga]Ga-DTPA[Ch-DE]_2_ and [^18^F]-Florbetapir in normal rats 2 min and 30 min post-injection

**Compound**	**%ID/g Brain**		**Ratio** **(2 min / 30 min)**
	**2 min**	**30 min**	
[^68^Ga]Ga-DTPA[Ch-DE]_2_	0.54 ± 0.34	0.26 ± 0.22	2.07
[^18^F]-Florbetapir	4.9 ± 0.99	1.65 ± 0.11	2.96,^†,50^

†: Reference 50

Figure 7 presents PET/CT imaging obtained 30 min after injection of [^68^Ga]Ga-DTPA[Ch-DE]_2_ and [^18^F]-Florbetapir in animals. Due to the lipophilicity of ^68^Ga-DTPA[Ch-DE]_2_ and florbetapir, both of them showed prominent liver uptake; however, florbetapir, due to its relatively higher lipophilicity, shows predominant excretion in the hepatobiliary pathway. In contrast, [^68^Ga]Ga-DTPA[Ch-DE]_2_ showed double excretion routes, hepatobiliary and renal, which is also the reason for the accumulation of activity in the kidney as reported for other ^68^Ga-chalcone-derived radiotracers.^34^ The kidney uptake may be related to polar metabolites of the ^68^Ga-DTPA[Ch-DE]_2_. As shown in Figure 7, both radiotracers [^68^Ga]Ga-DTPA[Ch-DE]_2_ and [^18^F]-Florbetapir showed detectable brain uptake in AD rats. One of the challenges of gallium-labeled radiopharmaceuticals is their limited ability to pass through the blood-brain barrier due to their inherent hydrophilic nature.^57-59^ In the current study, the chalcone dimer was used due to the placement of the DTPA in the middle, which, in addition to enabling labeling, also caused the addition of two lipophilic arms on both sides of the molecule, which increased their lipophilicity and the ability to enter the brain. Although the brain uptake of [^68^Ga]Ga-DTPA[Ch-DE]_2 _was comparatively lower compared to florbetapir, this small amount of brain entry is desirable and considered a success for gallium-labeled radiopharmaceuticals.

**Figure 7:**
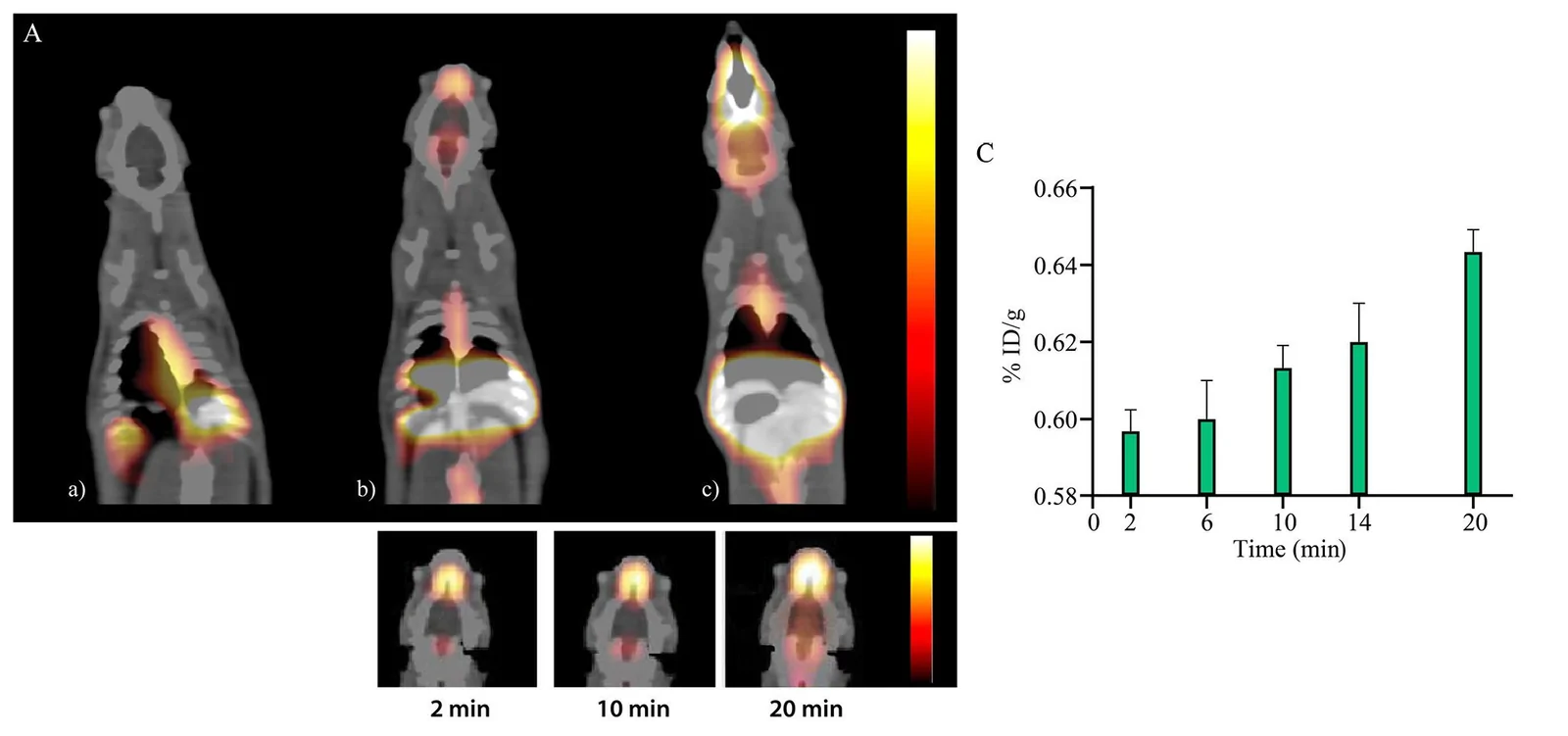
**A**) Fused PET/CT images taken 30 min p.i of [^68^Ga]Ga-DTPA[Ch-DE]_2_, **a**) normal, **b**) AD rat, **c**) Fused PET/CT images taken 30 min p.i of Florbetapir in AD rat for 10 min; **B**) Dynamic PET/CT images of AD rat up to 20 min p.i of [^68^Ga]Ga-DTPA[Ch-DE]_2_.C) Time-activity curve of dynamic pet imaging for up to 20 min p.i of [^68^Ga]Ga-DTPA[Ch-DE]_2_ in AD rat

 One of the most critical requirements for a successful brain imaging agent is the ability to penetrate well through the BBB, deliver a appropriate level of radioactive to the brain, and clear rapidly from normal, beta-amyloid-free areas, to give a good signal-to-noise ratio. Dynamic PET/CT imaging was performed for 20 min post-injection of [^68^Ga]Ga-DTPA[Ch-DE]_2 _in AD rats to evaluate blood-brain barrier (BBB) permeability and brain pharmacokinetics. The results of dynamic imaging in Alzheimer’s rats demonstrated an increase in brain uptake with a slow clearance rate (Figure 7B). The findings are evidence for the feasibility of [^68^Ga]Ga-DTPA[Ch-DE]_2_ to image Aβ aggregates in vivo.

###  Molecular Docking

 Docking studies were performed to evaluate the interactions of DTPA[Ch-DE]_2_, DTPA[Ch-DM]_2_, PIB, and florbetapir compounds as reference ligands with both the Aβ monomer and fibril. The obtained values of binding energies in the best-docked conformation (lowest energy values) were summarized in Table 2. DTPA[Ch-DE]_2_ revealed a strong binding affinity to Aβ42 fibril with a binding energy of -5.7 kcal/mol compared to DTPA[Ch-DM]_2 _at -5.2 kcal/mol, PIB at -5.0 kcal/mol, and florbetapir at -4.0 kcal/mol.

**Table 2:** Docking results of the best-docked conformation using AutoDock Vina Software

**Compounds**	**Binding affinity energy (kcal/mol)**
DTPA[Ch-DE]_2_ with Aβ monomer	-3.6
DTPA[Ch-DE]_2_ with Aβ fibril DTPA[Ch-DM]_2_ with Aβ fibril	-5.7 -5.2
Florbetapir (AV-45) with Aβ fibril	-4.0
PIB with Aβ fibril	-5.0

 DTPA[Ch-DE]_2_ demonstrated a strong binding mode with Aβ fibrils using a combination of the hydrophobic interactions with several amino acid residues, including Gly37, Gly38, Val36, Val39, Val40, Met35, Leu17, and Phe19, along with a hydrogen bond formed with Gly37 (Figure 8). These findings indicate that DTPA[Ch-DE]_2_ has a good binding capacity towards Aβ plaques.

**Figure 8:**
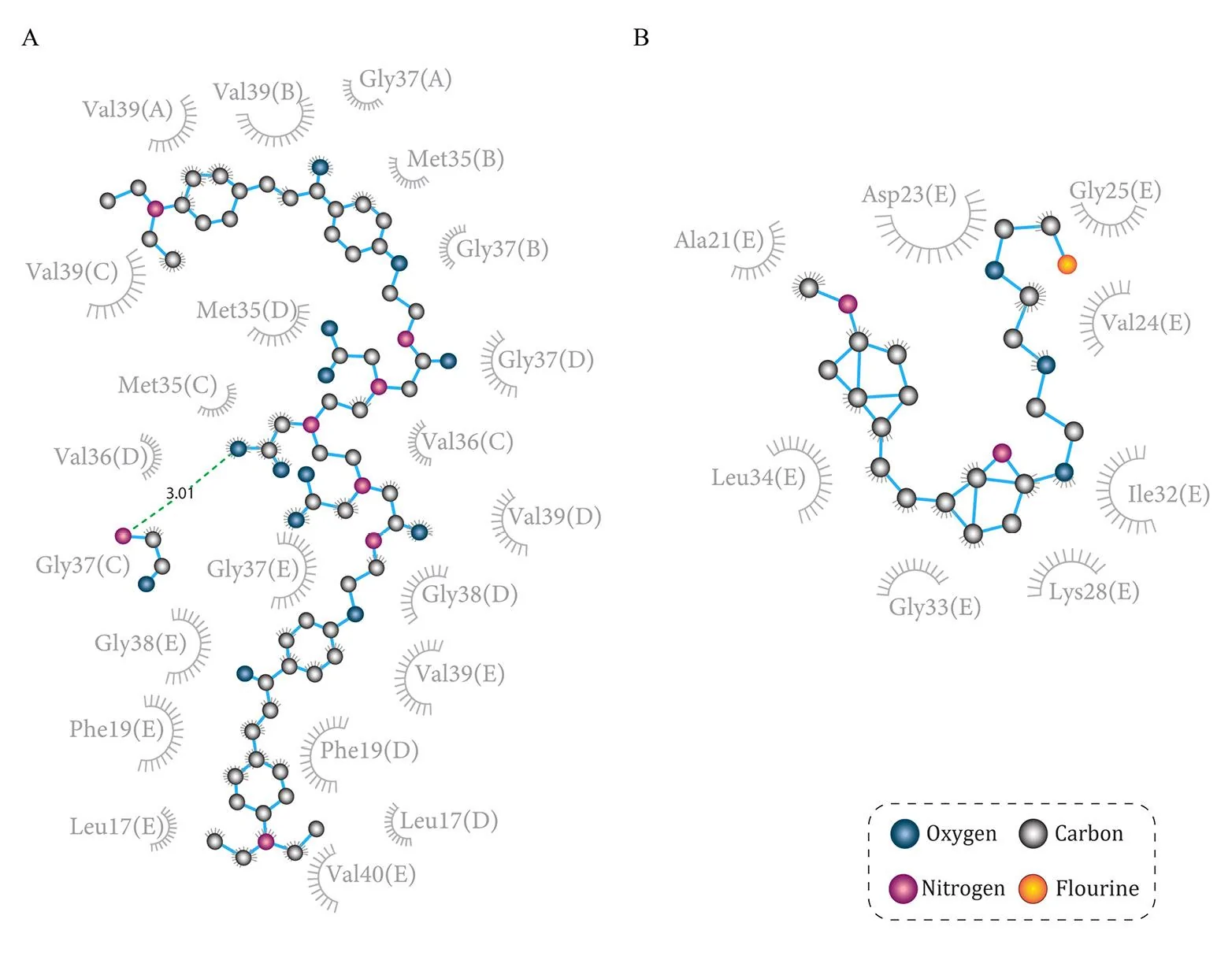
2D schematic representation of the interactions: **A**) DTPA[Ch-DE]_2_, and B) AV-45 with Aβ42 pentameric fibril (2BEG)

###  Molecular Dynamics Simulation

 The thermodynamic and structural behavior of ligand-receptor complexes was analyzed through MD simulations. The interaction energies, force distributions, and structural dynamics were compared for the AV-45 and DTPA[Ch-DE]_2_. Figure 9A presents the average non-bonded interaction energies—comprising electrostatic and van der Waals components—between receptor residues and ligands across three independent simulations. DTPA[Ch-DE]_2_ consistently exhibited more favorable (i.e., more negative) binding energies than AV-45, indicating higher thermodynamic stability. These pronounced energy minima suggest potential binding hotspots and reflect the enhanced electrostatic complementarity of the DTPA[Ch-DE]_2_ with the receptor.Figure 9B illustrates the force distribution across receptor residues interacting with the DTPA[Ch-DE]_2_. Red regions (-8 to -5 kcal/mol·Å) highlight residues forming strong attractive interactions. The RoG analysis in Figure 9 Cevaluates the structural compactness of the receptor in three states: the unbound receptor, the receptor bound to the control compound (AV-45), and the receptor bound to the DTPA[Ch-DE]_2_. The results indicate that both ligands have a similar effect on the RoG of the receptor, as their trajectories closely overlap over the last 100 ns of the simulation period. As can be seen, for the unbound receptor, the RoG fluctuates between ~14.1–14.7 Å, reflecting the inherent flexibility of the protein in its free state. However, in the presence of the ligands (both DTPA[Ch-DE]_2_ and AV-45 compound), the RoG values reduce slightly (~0.2 Å) and remain within the same range with no significant deviation. This suggests that AV-45 binding does not induce major structural compaction or expansion.

**Figure 9:**
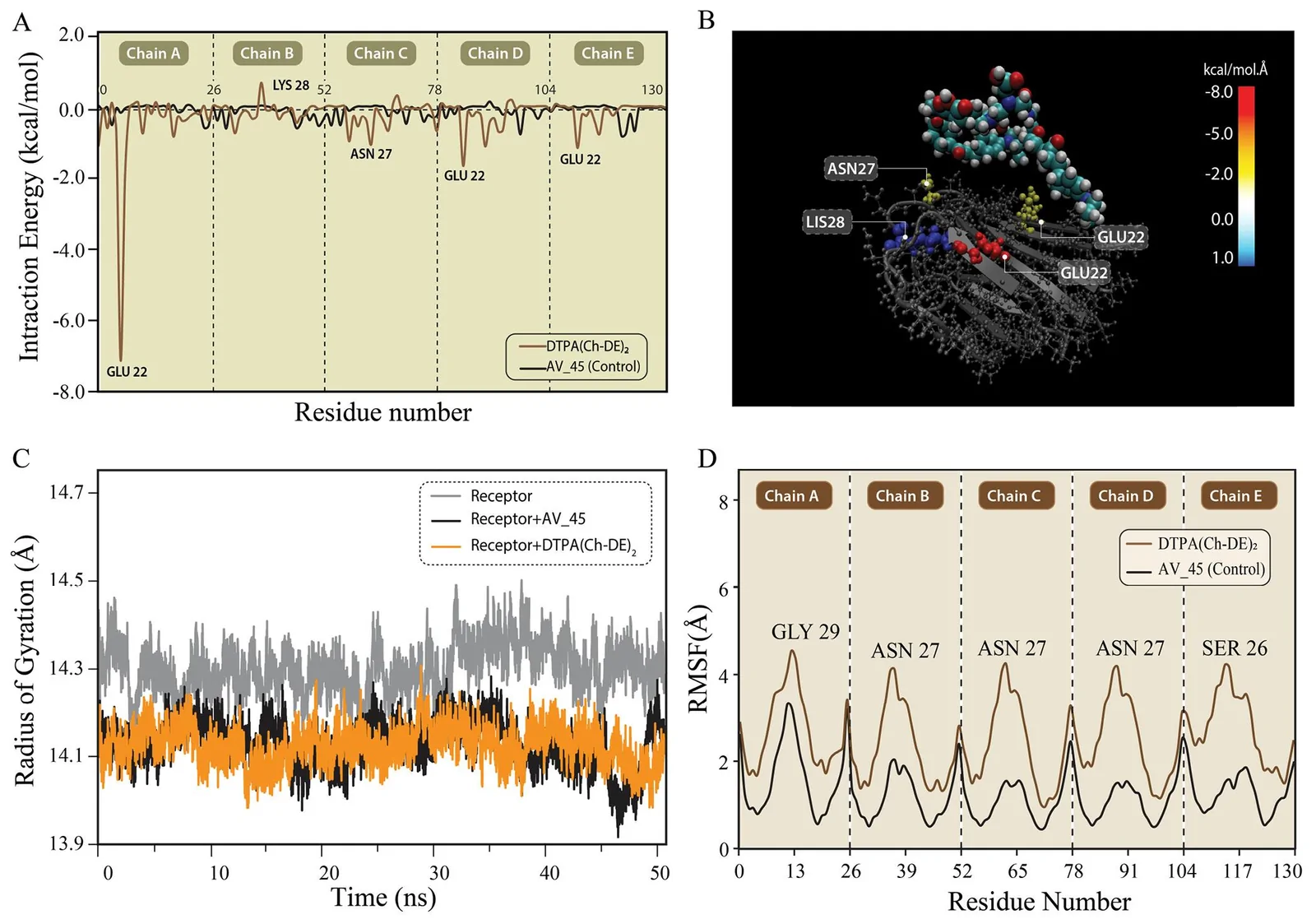
MD simulation results, **A**) Total non-bonded interaction energies (kcal/mol) between ligands and receptor residues, averaged across three replicates; **B**) Spatial force map between the DTPA[Ch-DE]_2_ and receptor residues. Red and blue denote attractive and repulsive forces, respectively;** C**) Time-dependent changes in the radius of gyration (RoG) for the receptor alone (gray), receptor with AV_45 control (black), and receptor with the DTPA[Ch-DE]_2_ (orange). The plot illustrates structural compactness over a 100 ns simulation period, with RoG values (Å) on the y-axis and simulation time (ns) on the x-axis.;** D**) Root Mean Square Fluctuation (RMSF) analysis of Aβ42 fibril chains (A–E) for the DTPA[Ch-DE]_2_ compared to the control (AV-45). The plot highlights fluctuations at specific residues, including Gly 29, Asn 27, and Ser 26, with residue numbers indicated along the x-axis. RMSF values (y-axis) reflect the flexibility of the protein backbone across the evaluated regions

 The RMSF analysis (Figure 9D) reveals critical differences in local flexibility between the AV-45-bound and DTPA[Ch-DE]_2_-bound receptor systems, despite their similar effects on global compactness (RoG: Figure 9C). These residue-specific variations provide valuable insights into ligand-induced dynamic changes. Both compounds show comparable RMSF profiles for most of the receptor structure (residues 13-130), with fluctuations typically below 2 Å, indicating generally stable secondary structural elements. However, a moderate difference was observed at residue Gly29, where DTPA[Ch-DE]₂ showed higher RMSF (~6 Å) compared to AV-45 (~4 Å). Of note, Gly29 resides within the naturally flexible turn region (residues 25–29) of the Aβ fibril, and elevated RMSF in this loop does not necessarily indicate enhanced binding. The adjacent region (residues 26-27, containing Asn27 and Ser26) also displays increased mobility with DTPA[Ch-DE]_2_, though to a lesser degree. Moreover, molecular docking and MD simulations indicate that DTPA[Ch-DE]_2_ exhibits superior binding performance compared to AV-45 florbetapir. This is evidenced by stronger interaction energies, favorable force distributions, and enhanced receptor stabilization (Figure 9). The results of force distribution highlight residues involved in strong attractive interactions Aβ fibril with the DTPA[Ch-DE]_2_. For instance, Glu22 likely engages in π-π stacking or hydrophobic interactions, contributing significantly to binding affinity. Tryptophan, due to its rigid structure, may restrict ligand motion, enhancing complex stability. Additionally, polar residues such as Glu22 may form hydrogen bonds, aiding adaptive ligand orientation. In contrast, Lys28 exhibits weak repulsive forces (~0–1 kcal/mol·Å), likely due to electrostatic or steric effects. These forces might result from the carboxylate group of Lys28 interacting unfavorably with negatively charged or bulky moieties on the ligand.The RoG analysis demonstrates that both the control (AV-45) and DTPA[Ch-DE]_2_ do not significantly alter the receptor’s structural compactness. The lack of significant RoG variation suggests that the binding of these ligands primarily influences local interactions (e.g., side-chain rearrangements or binding pocket dynamics) rather than large-scale structural reorganization. Since DTPA[Ch-DE]_2_ mimics the effect of the control (AV-45), it may share a similar binding mode or induce comparable dynamic restraints on the receptor.

 These computational results support the hypothesis that DTPA[Ch-DE]_2_ maintains the receptor’s native conformational flexibility - a feature that may be functionally advantageous for its intended therapeutic role. To further characterize these effects, additional investigations were performed on local structural perturbations through RMSF analysis. The RMSF analysis provides residue-level insights into the dynamic changes induced by ligand binding. This higher-resolution analysis complements the global perspective offered by the RoG measurements and helps identify specific regions of the receptor that may be functionally important for compound optimization.

 The notable point in the RMSF analysis is that the multi-chain structure shows consistent RMSF patterns across chains (A-E), suggesting the observed flexibility changes are inherent to the protein’s structural response rather than chain-specific artifacts. From a structural and functional perspective, RMSF analysis revealed that both DTPA[Ch-DE]₂ and AV-45 maintain generally similar flexibility profiles across most residues of the Aβ fibril (residues 13–130), with fluctuations typically below 2 Å. However, a notable difference was observed at Gly29 and adjacent residues (Ser26, Asn27), where DTPA[Ch-DE]₂ exhibited moderately higher RMSF (~6 Å) compared to AV-45 (~4 Å). Structurally, Gly29 is located within the turn region connecting the β-strands of the Aβ fibril (residues 25–29). This segment is inherently flexible due to the absence of stabilizing side-chain interactions and its role in allowing β-strand registration. Therefore, the increased RMSF observed for DTPA[Ch-DE]₂ does not necessarily indicate superior binding. Instead, it likely reflects that DTPA[Ch-DE]₂ engages the fibril through interactions that permit—or do not restrict—the natural conformational sampling of this loop region.

 The preserved global stability (RoG) alongside localized flexibility changes implies that the DTPA[Ch-DE]_2_ maintains overall receptor architecture while permitting specific functional motions, potentially generating distinct dynamic signatures that could influence allosteric communication. Notably, while both ligands maintain similar global receptor stability (RoG, Figure 9C), their RMSF profiles reveal key differences: AV-45 stabilizes specific local regions more effectively, whereas DTPA[Ch-DE]_2_ allows greater local flexibility in particular structural elements, particularly at Gly29 and adjacent residues, suggesting divergent binding mechanisms despite comparable overall structural impacts. The combination of preserved global structure with targeted local flexibility modifications suggests DTPA[Ch-DE]_2_ may offer novel functional properties while maintaining the receptor’s overall architectural integrity. These differences likely reflect unique binding interactions of DTPA[Ch-DE]_2_ that could be leveraged for therapeutic optimization.

## Conclusion

 [^68^Ga]Ga-DTPA[Ch-DE]_2_ was successfully prepared with high radiochemical yield and purity. The radiotracer exhibited high stability against HSA and good binding affinity to Aβ aggregations in vitro binding assay and the brain sections of AD rats. The findings of biodistribution and imaging studies highlighted the favorable pharmacokinetic behavior of [^68^Ga]Ga-DTPA[Ch-DE]_2,_ such as its ability to pass through BBB and slow washout from the brain of AD rats over time. Molecular docking and MD simulation confirmed the binding affinity and key interactions involved between DTPA[Ch-DE]_2_ and Aβ fibrils. These findings demonstrate that [^68^Ga]Ga-DTPA[Ch-DE]_2_ could be a promising PET radiotracer for imaging Aβ plaques in AD.

## Competing Interest

 The authors declare no conflict of interest.

## Data Availability Statement

 All data will be available under request.

## Ethical Approval

 The study was approved by the ethics committee of Tehran University of Medical Sciences (Tehran, Iran) under Approval Code: IR.TUMS.TIPS.REC.1398.172.

## Supplementary Files

Supplementary file contains ^1^H NMR, FT-IR, and HRMS-ESI spectra as well as HPLC chromatograms of synthesized compounds (Figures S1-S9).
