# Supplementary material for: Design and Evaluation of a Chalcone-Based Dimeric Scaffold Targeting Amyloid-Beta Plaques for Early Detection of Alzheimer’s Disease: Synthesis, Radiolabeling, In Vivo Studies, and Integrated Molecular Docking and Dynamics Simulation
Source: Adv Pharm Bull. 2026 May 19;16(2):416–31. doi: 10.34172/apb.46659 (PMC13745414; doi:10.34172/apb.46659)
Supplement: Supplementary file 1 [file apb-16-416-s001.pdf]

Adv Pharm Bull 2026;16(2):416-S1
doi:10.34172/apb.46659
https://apb.tbzmed.ac.ir

Supplementary Materials

List of contents

Figure S1. ^{1}H NMR spectrum of *tert*-Butyl 2-bromoethylcarbamate (Compound 1)

Figure S2. ^{1}H NMR spectrum of Para-diethylaminobenzaldehyde (Compound 2)

Figure S3. ^{1}H NMR spectrum of (Z)-3-(4-(diethylamino)phenyl)-1-(4-hydroxyphenyl) prop-2-en-1-one (Compound 3)

Figure S4. MS-EI spectrum of (Z)-3-(4-(diethylamino)phenyl)-1-(4-hydroxyphenyl) prop-2-en-1-one (Compound 3)

Figure S5. ^{1}H NMR spectrum (Z)-tert-butyl-2-(4-(3-(4-(diethylamino)phenyl)acryloyl)-phenoxy)ethyl)carbamate (Compound 4)

Figure S6. ^{1}H NMR spectrum (Z)-1-(4-(2-aminoethoxy) phenyl)-3-(4-(diethylamino) phenyl) prop-2-en-1-one (Compound 5)

Figure S7. HPLC profile of DTPA[Ch-DE]$_2$ (Compound 6)

Figure S8. Mass spectrum of DTPA[Ch-DE]$_2$ (Compound 6)

Figure S9. FT-IR spectrum of (Z)-1-(4-(2-aminoethoxy) phenyl)-3-(4-(diethylamino) phenyl) prop-2-en-1-one (Compound 5), and DTPA[Ch-DE]$_2$ (Compound 6)

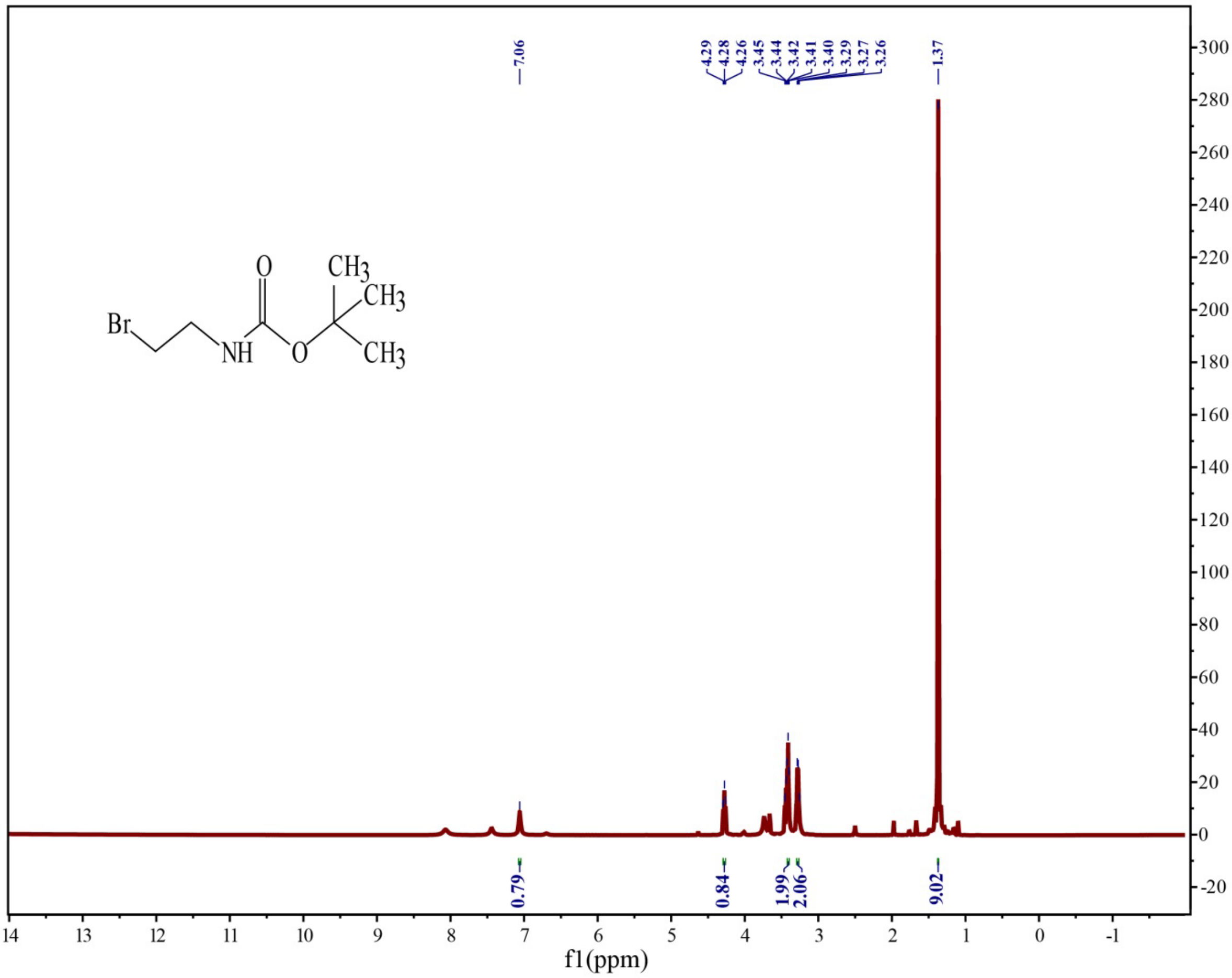

Figure S1. ^{1}H NMR spectrum of *tert*-Butyl 2-bromoethylcarbamate (Compound 1).

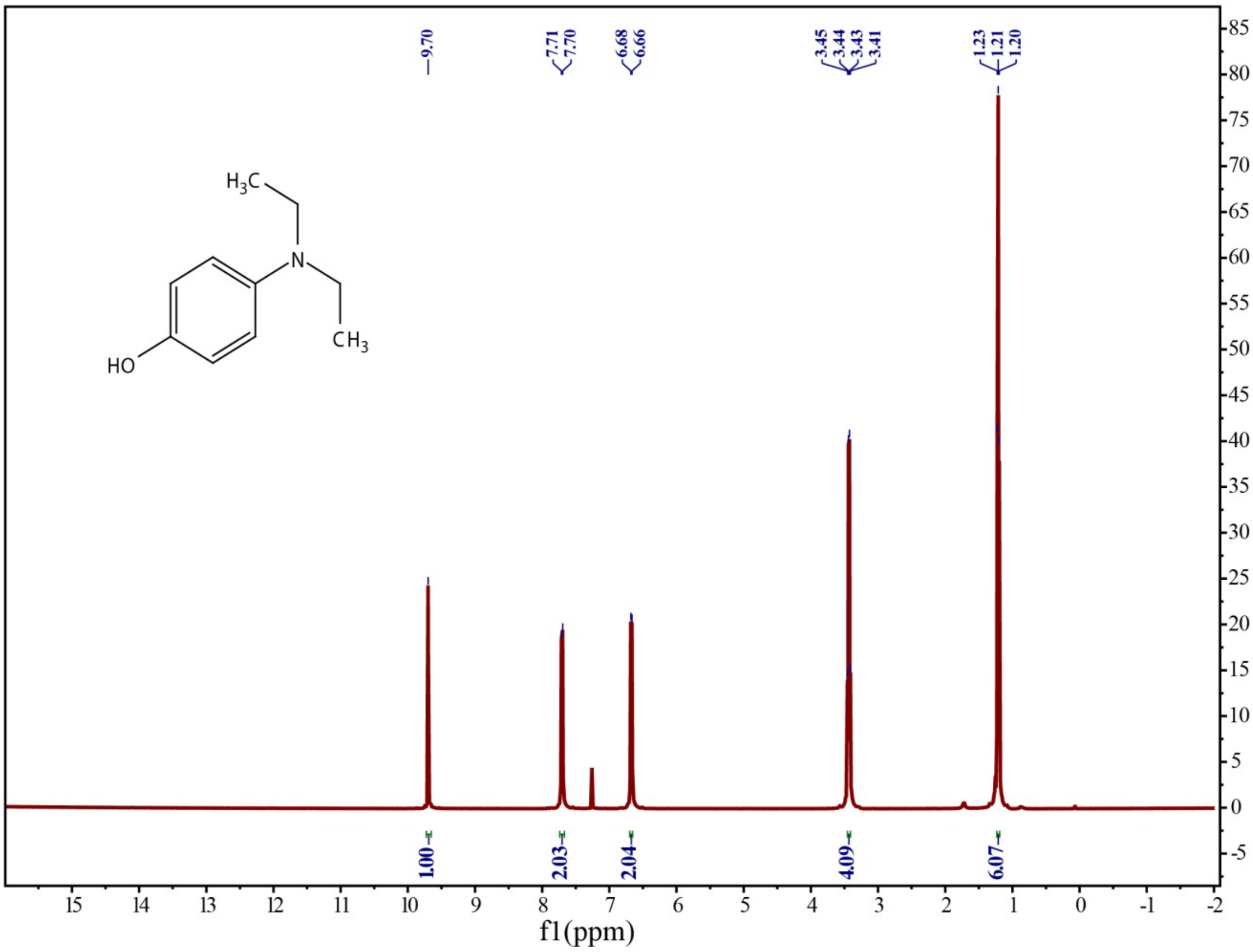

Figure S2. ^{1}H NMR spectrum of Para-diethylaminobenzaldehyde (Compound 2).

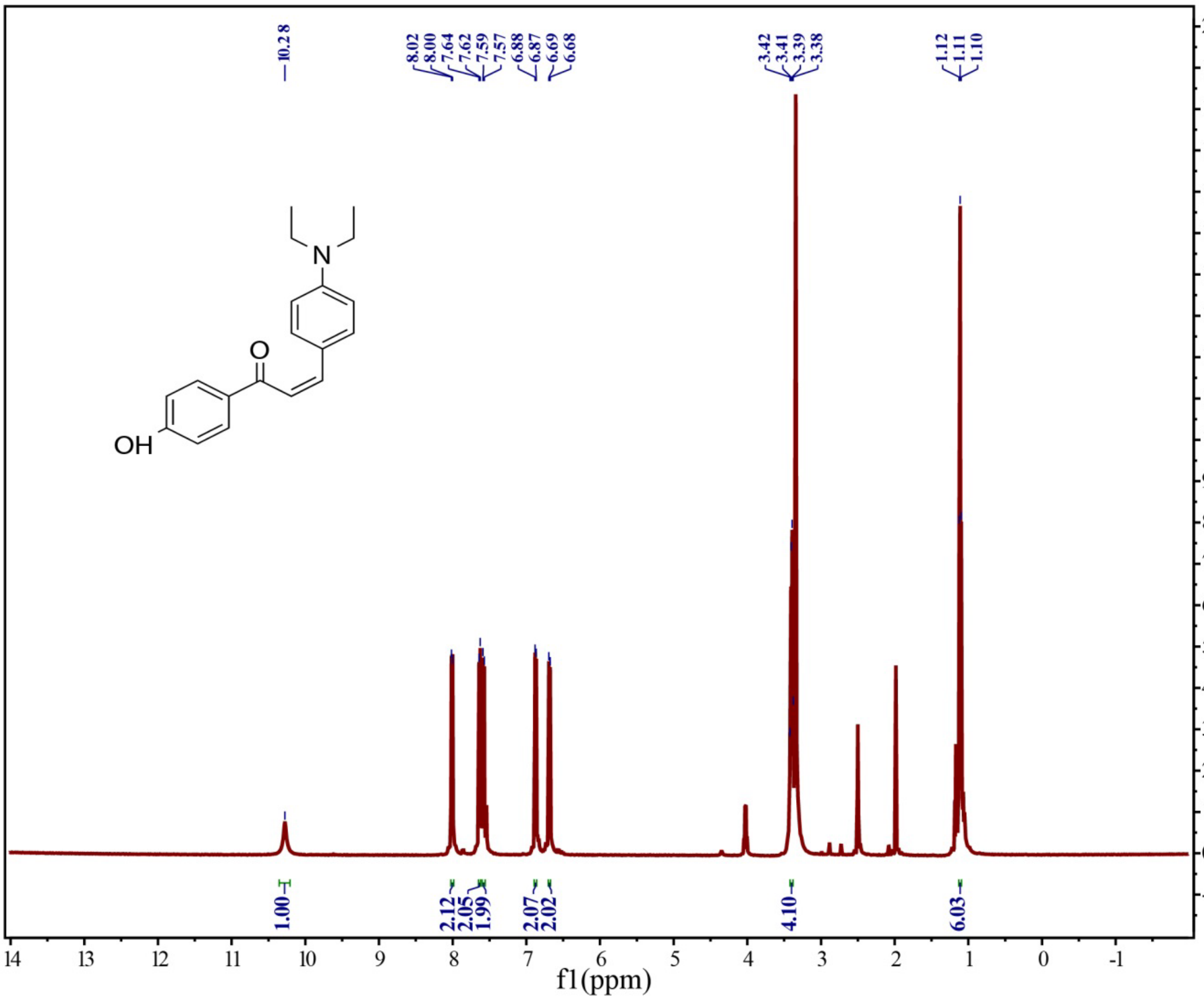

Figure S3. ^{1}H NMR spectrum of (Z)-3-(4-(diethylamino)phenyl)-1-(4-hydroxyphenyl) prop-2-en-1-one (Compound 3).

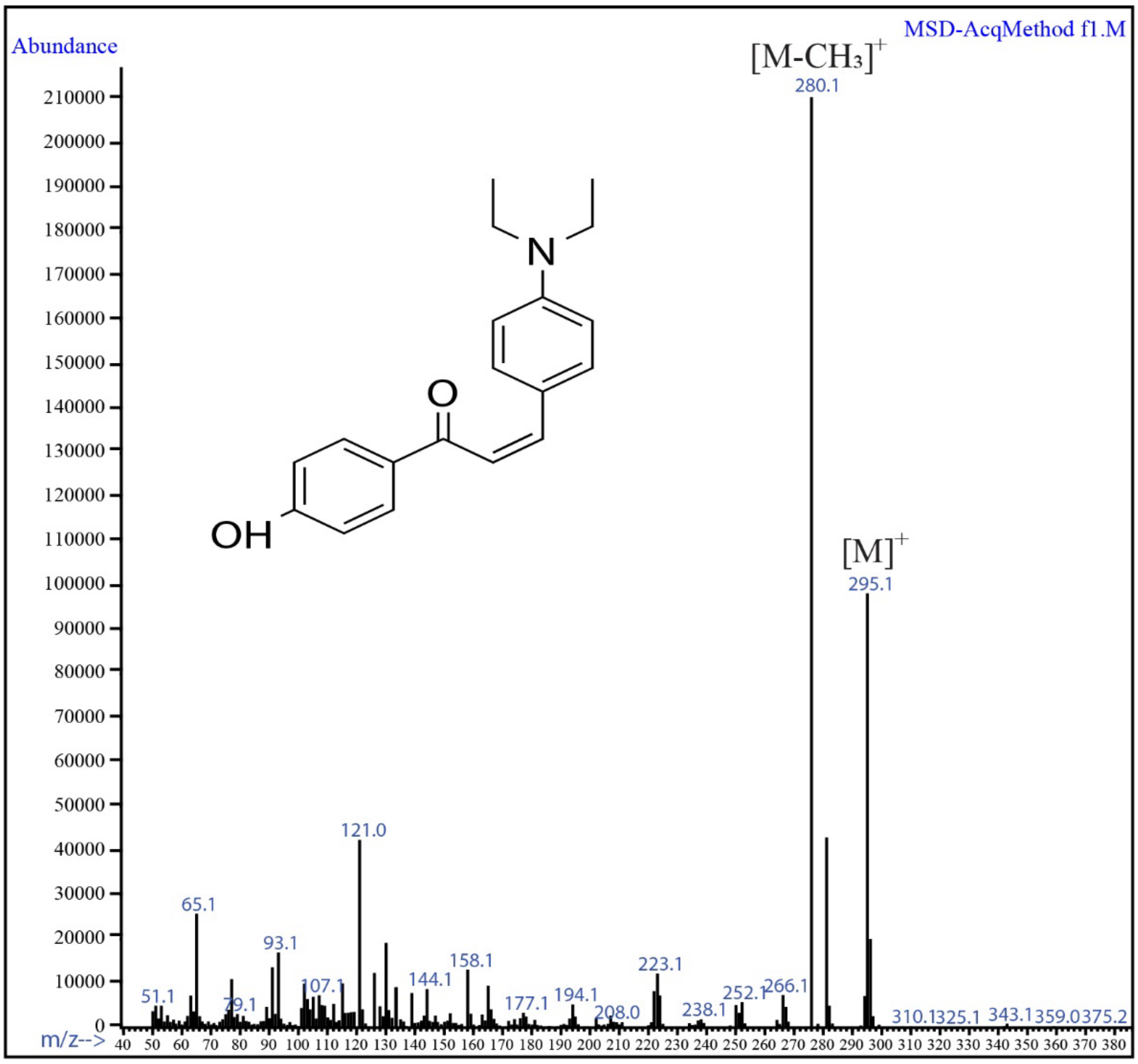

Figure S4. MS-EI spectrum of (Z)-3-(4-(diethylamino)phenyl)-1-(4-hydroxyphenyl) prop-2-en-1-one (Compound 3).

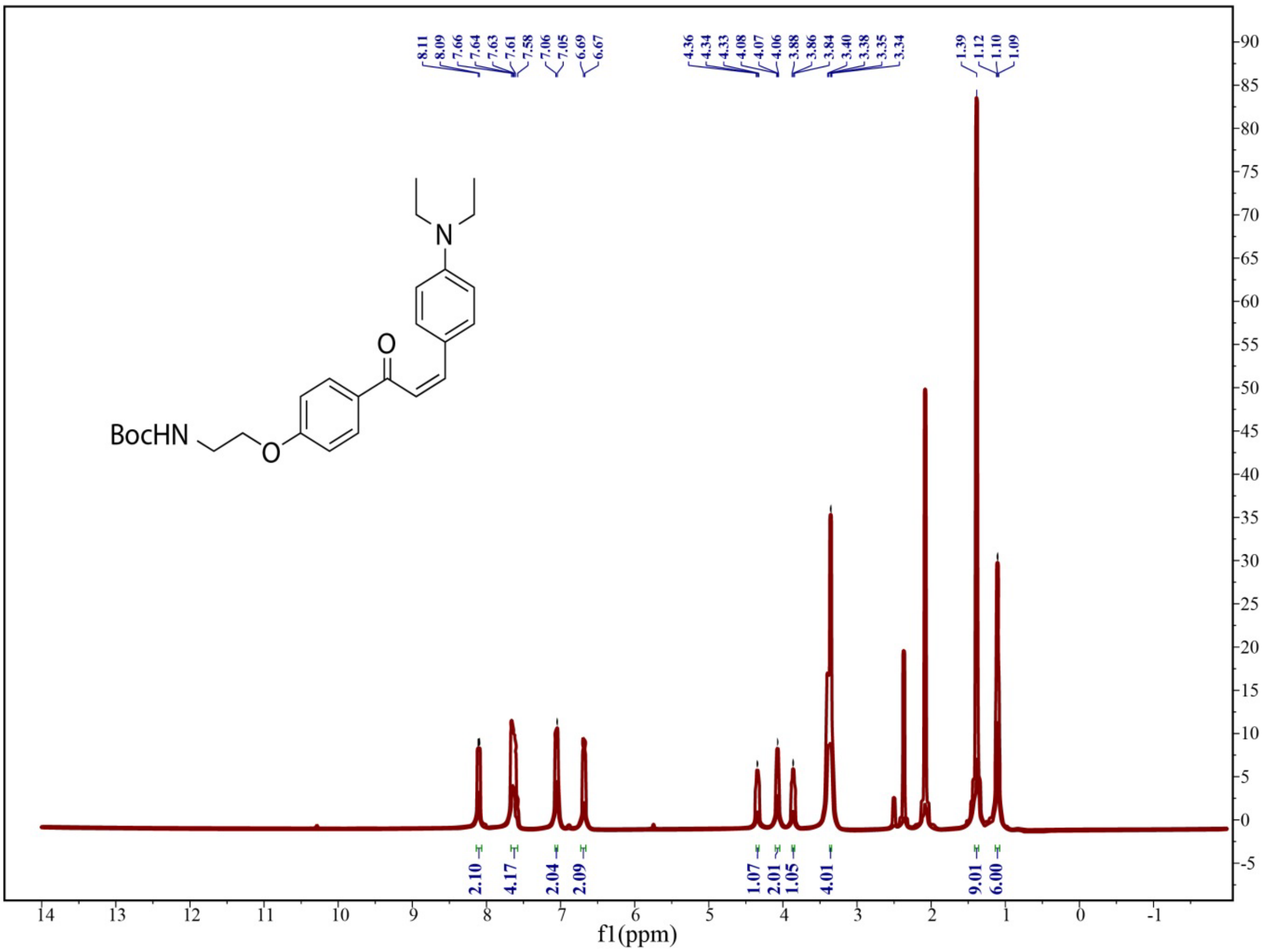

Figure S5. ^{1}H NMR spectrum (Z)-tert-butyl-2-(4-(3-(4-(diethylamino)phenyl)acryloyl)-phenoxy)ethyl)carbamate (Compound 4).

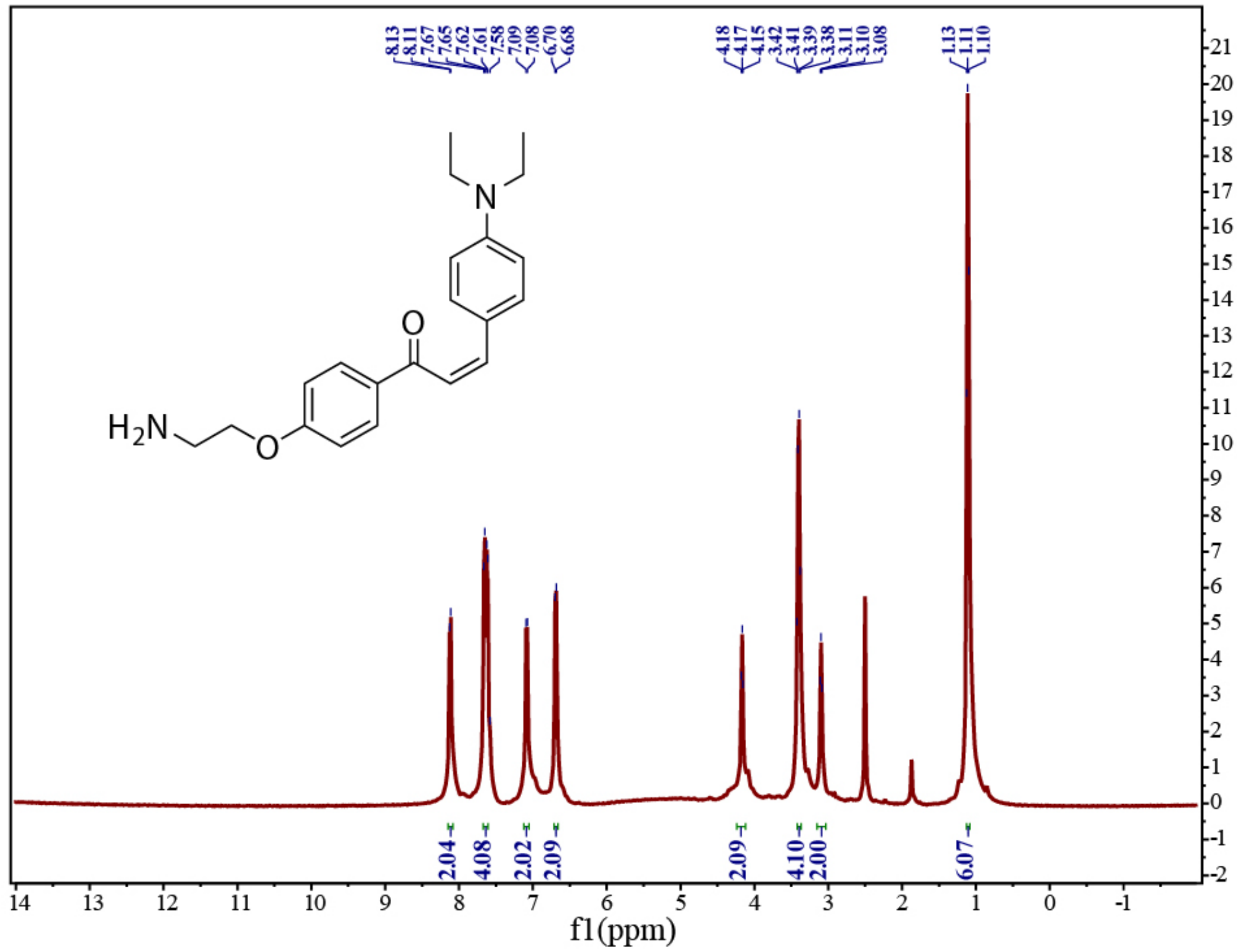

Figure S6. ^{1}H NMR spectrum (Z)-1-(4-(2-aminoethoxy) phenyl)-3-(4-(diethylamino) phenyl) prop-2-en-1-one (Compound 5).

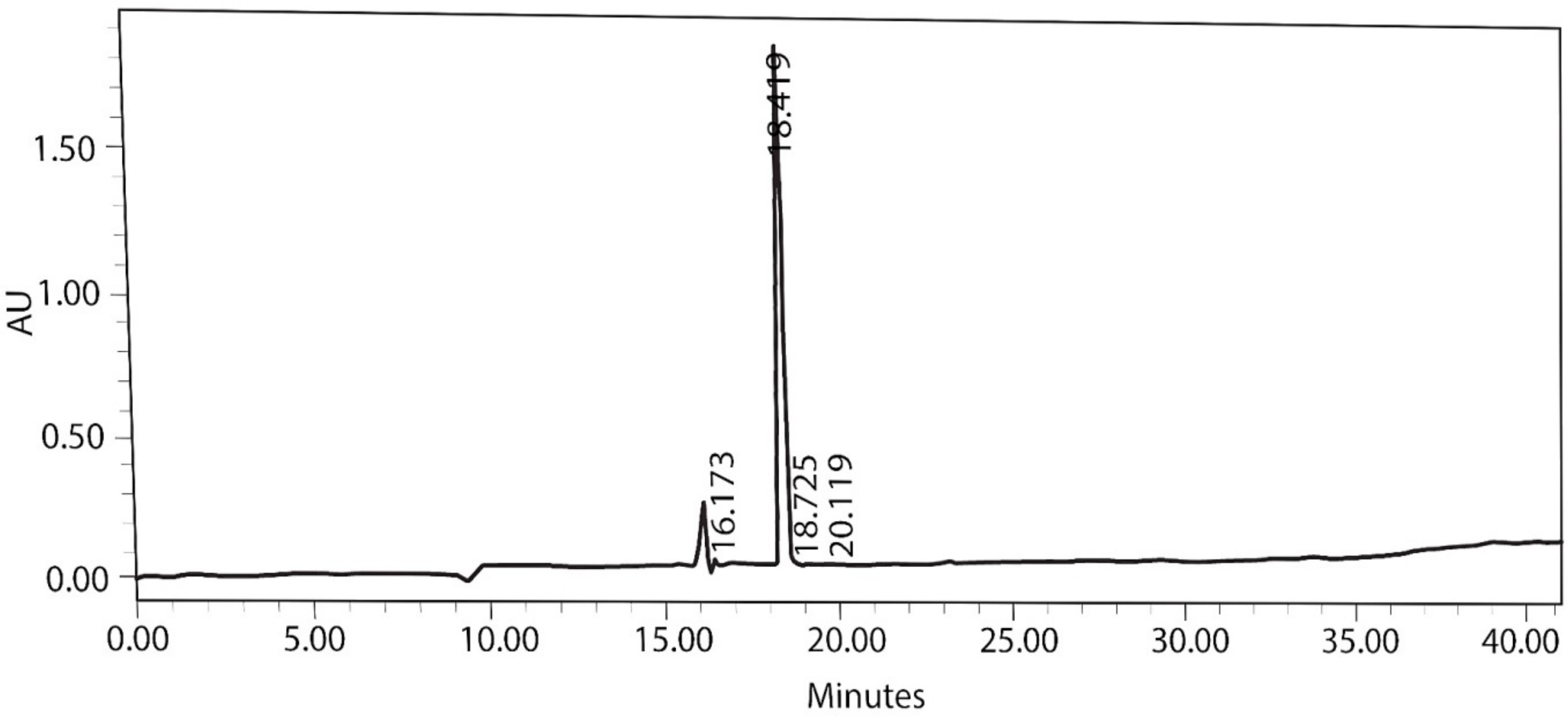

Figure S7. HPLC profile of DTPA[Ch-DE]$_2$ (Compound 6).

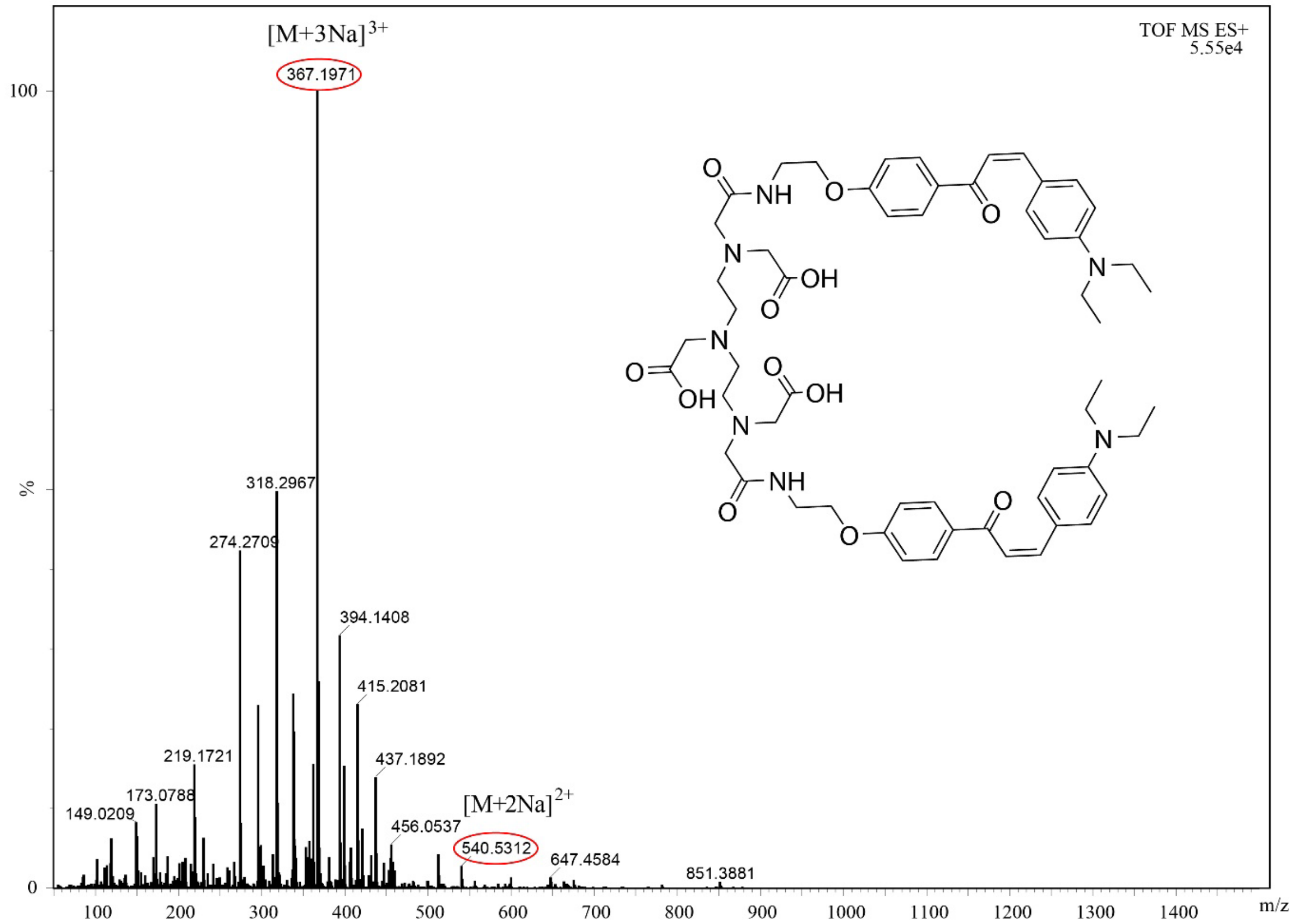

Figure S8. Mass spectrum of DTPA[Ch-DE]2 (Compound 6).

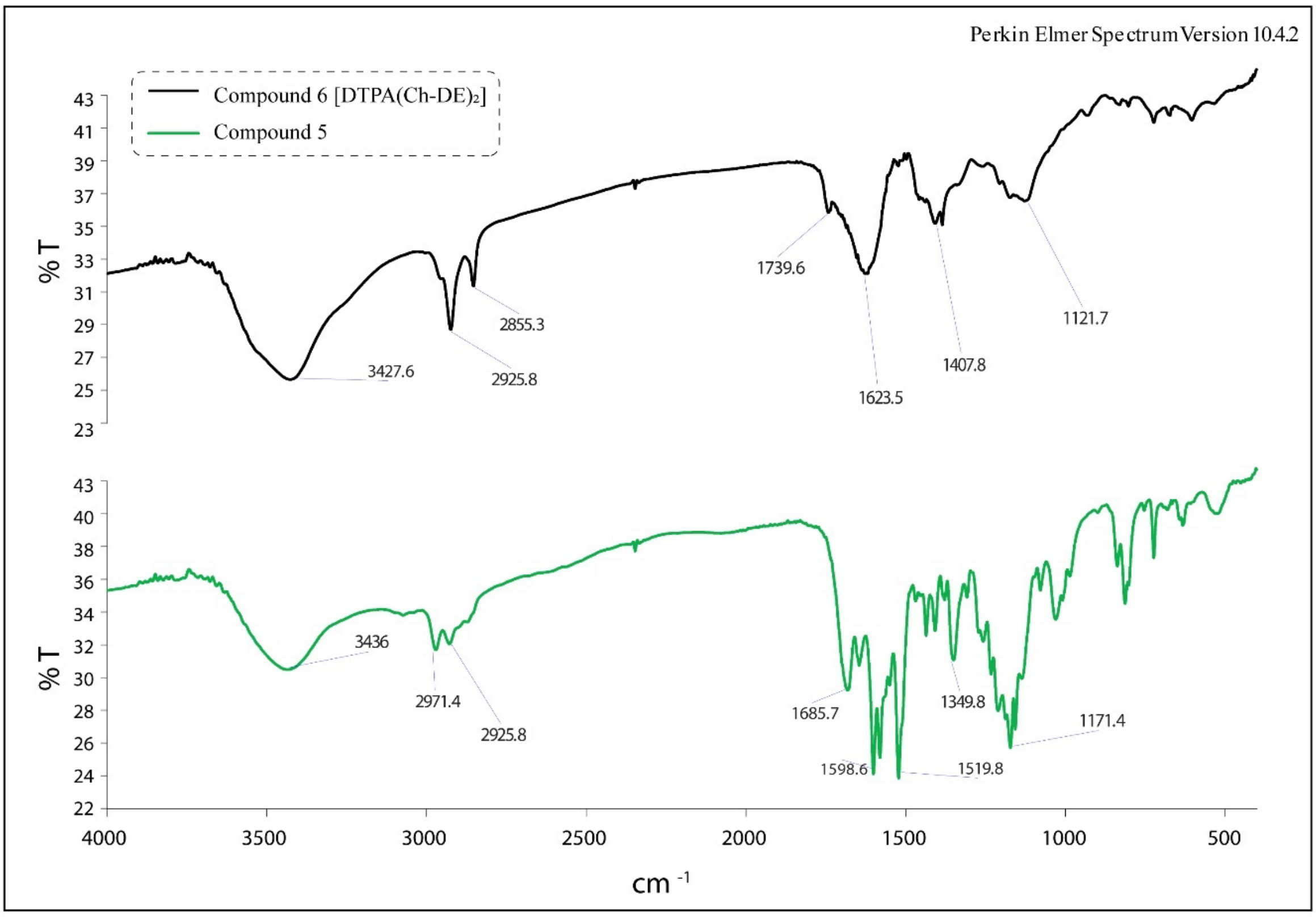

Figure S9. FT-IR spectrum of (Z)-1-(4-(2-aminoethoxy) phenyl)-3-(4-(diethylamino) phenyl) prop-2-en-1-one (Compound 5), and DTPA[Ch-DE]$_2$ (Compound 6).
